# Supplementary material for: Oxophilic Ce single atoms-triggered active sites reverse for superior alkaline hydrogen evolution
Source: Nat Commun. 2024 Jan 10;15:448. doi: 10.1038/s41467-024-44721-5 (PMC10782026; doi:10.1038/s41467-024-44721-5)
Supplement: Supplementary file 1 — Supplementary Information [file 41467_2024_44721_MOESM1_ESM.pdf]

Supplementary Information

Oxophilic Ce single atoms-triggered active sites reverse for superior alkaline hydrogen evolution

Fengyi Shen,[1,#] Zhihao Zhang,[2,#] Zhe Wang,[1,#] Hao Ren,[1] Xinhu Liang,[1] Zengjian Cai,[1] Shitu Yang,[1] Guodong Sun,[1] Yanan Cao,[1] Xiaoxin Yang,[1] Mingzhen Hu,[1,2,*] Zhengping Hao,[2,*] and Kebin Zhou[1,2,*]

[1]School of Chemical Sciences, [2]National Engineering Laboratory for VOCs Pollution Control Material & Technology, Research Center for Environmental Material and Pollution Control Technology, University of Chinese Academy of Sciences, Beijing, 100049, PR China.

[#]These authors contributed equally to this work.

**E-mail: humingzhen12@ucas.ac.cn; zphao@ucas.ac.cn; kbzhou@ucas.ac.cn*

Inventory of Supporting Information

Supplementary Figure S1 to S40

Supplementary Table S1 to S5

Supplementary Reference 1-117

Supplementary Figures

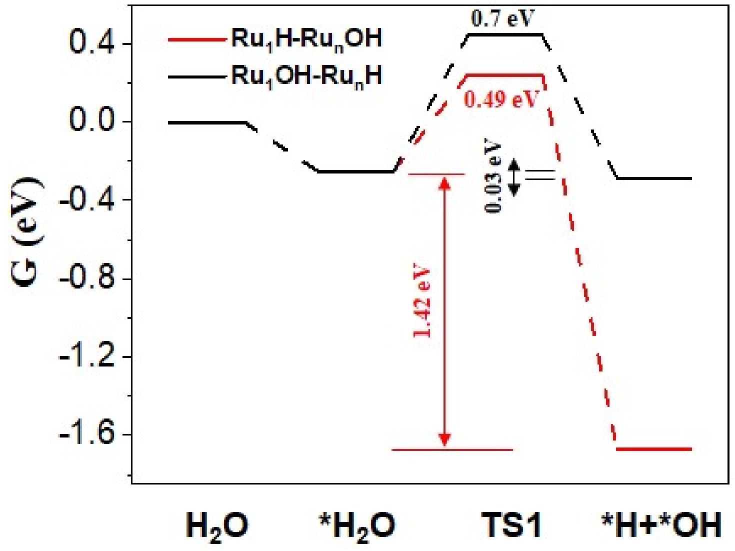

Figure S1. The Gibbs free energy diagrams for water dissociation via the Ru_1OH-Ru_nH route and via the Ru_1H-Ru_nOH route, respectively. Water dissociation theoretically had two possible patterns on the dual Ru_1-Ru_n site (n=13 in current work, corresponding to a Ru_n nanocluster of about 1 nm): i) the OH group bonded with Ru_1 while the H atom bonded with Ru_n (Ru_1OH-Ru_nH route); and ii) the H atom bonded with Ru_1 while the OH group bonded with Ru_n (Ru_1H-Ru_nOH route). As displayed in Figure S1, the Gibbs free energy barrier for water dissociation of the Ru_1H-Ru_nOH route was 0.49 eV, which was markedly lower than that of the Ru_1OH-Ru_nH route (up to 0.7 eV), unveiling the Ru_1H-Ru_nOH route was the more kinetically favored reaction pathway for water dissociation. On the other hand, it was found that water dissociation of the Ru_1H-Ru_nOH route was exothermic by 1.42 eV, while the water dissociation of the Ru_1OH-Ru_nH route was exothermic by only 0.03 eV, showing a 47-fold difference between them. This result further uncovered the more favorable thermodynamics for water dissociation via the Ru_1H-Ru_nOH route relative to that via the Ru_1OH-Ru_nH route. Therefore, driven by the more beneficial reaction kinetics and thermodynamics of the Ru_1H-Ru_nOH route, H would be bonded with the Ru_1 and OH would be bonded with the Ru_n after water dissociation, which made further hydrogen evolution occur on the Ru_1 side of the dual Ru_1-Ru_n site.

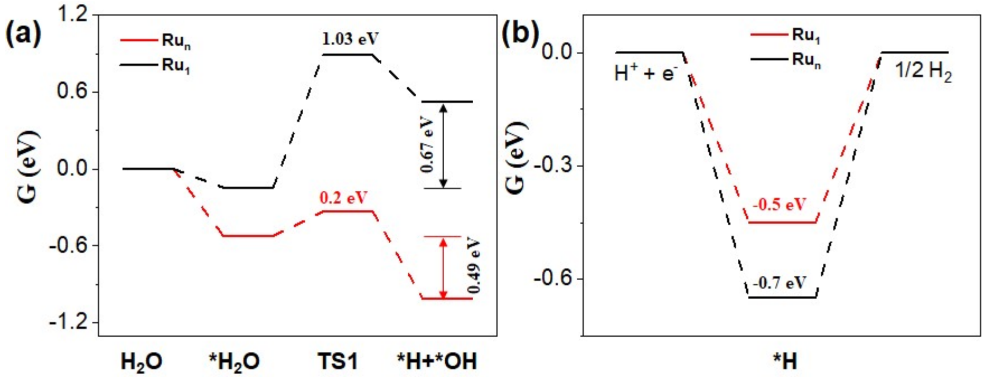

Figure S2. The DFT calculations for water dissociation and H adsorption over the Ru single atom and Ru$_n$ nanocluster, respectively. (a) The Gibbs free energy diagrams for water dissociation over the Ru single atom and over the Ru$_n$ nanocluster, respectively. (b) The H adsorption energies on the Ru single atom and on the Ru$_n$ nanocluster, respectively. As illustrated in Figure S1a, the Ru single atom (Ru$_1$) exhibited a particularly high Gibbs free energy barrier for water dissociation (up to 1.03 eV) while the value of the Ru$_n$ nanocluster (n=13 in our case, corresponding to a Ru$_n$ nanocluster of about 1 nm) was only 0.2 eV. In addition, water dissociation over the Ru$_n$ nanocluster was the thermodynamically favorable exothermic process with an exothermic energy of 0.49 eV. Whereas, it was endothermic by 0.67 eV on the Ru$_n$ nanocluster for dissociating water molecules. These results suggested that the Ru$_n$ nanoclusters were much more efficient for disassociating water than the Ru single atoms. On the other hand, the H adsorption energy on the Ru$_n$ nanocluster was -0.5 eV (Figure S2b), close to the optimal value (0 eV), while the H adsorption energy on the Ru single atom was -0.7 eV. These results revealed that the Ru$_n$ nanoclusters were more active for hydrogen evolution than the Ru single atoms both in terms of the energy barrier for water dissociation and the H adsorption energy.

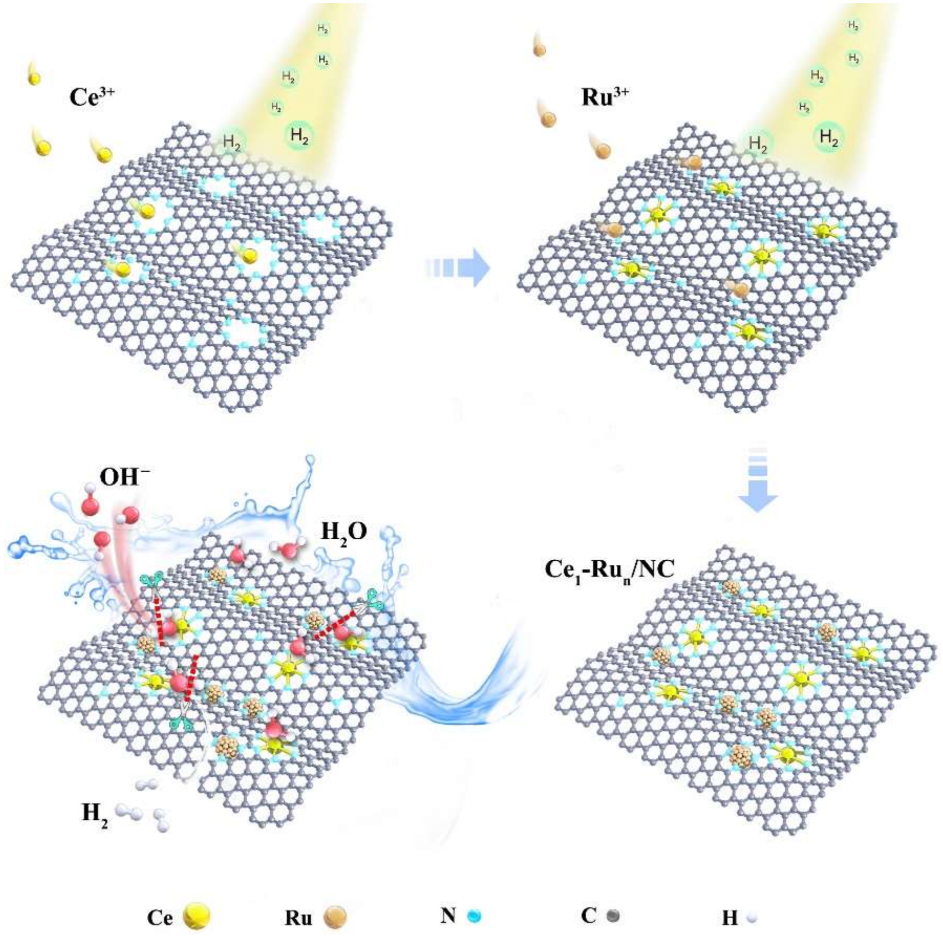

Figure S3. Schematic illustration of the synthetic process for the Ce_1-Ru_n/NC catalyst. To be noted, the Ce^{3+} species were firstly introduced to the NC support and then reduced by hydrogen at 700 °C for 2 hours (see methods of main text). After which, the uniformly dispersed CeO_2 was formed on the NC support (CeO_2/NC) and then etched to Ce single atoms (Ce_1/NC) with acid treatment (pH=1). Subsequently, the Ru^{3+} cations were introduced to the Ce_1/NC via impregnation and further reduced with hydrogen at 250 °C for 2 hours, forming the Ce_1-Ru_n/NC catalyst.

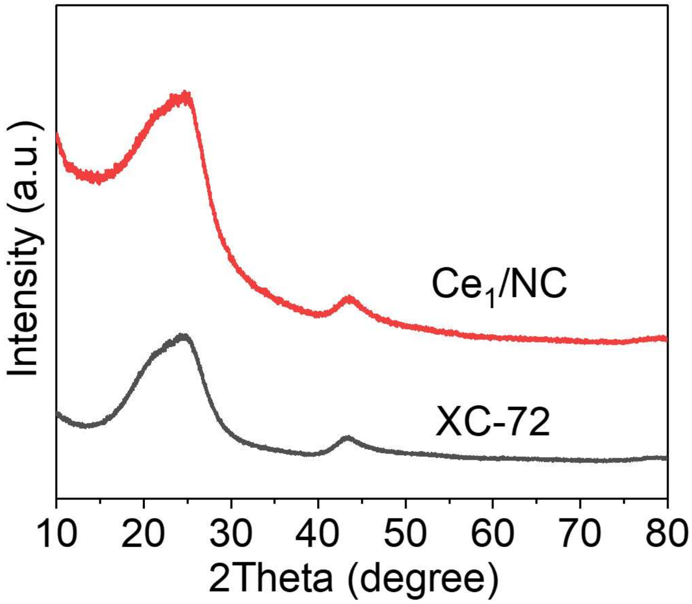

Figure S4. The XRD patterns of the Ce_1/NC catalyst and the pure XC-72 support. It was demonstrated that no XRD peaks were found for the Ce_1/NC catalyst except for the broad diffraction peaks of XC-72 carbon support in it.[1]

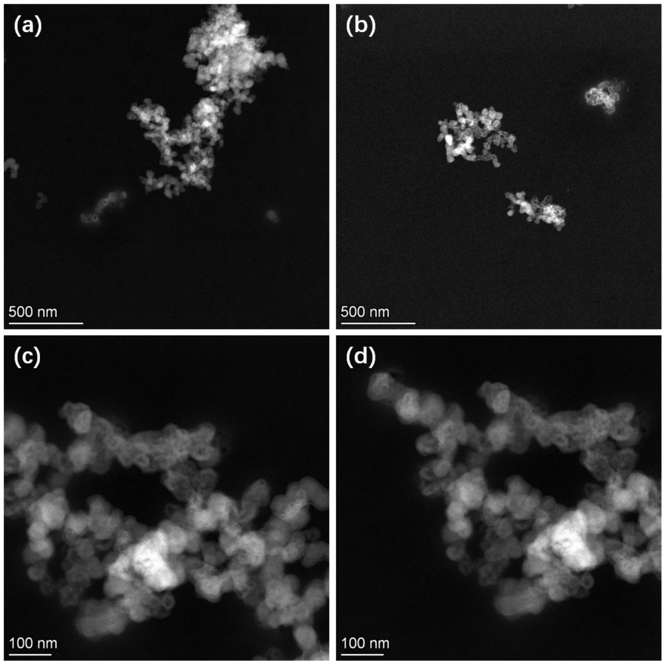

Figure S5. Microscopic measurements of the Ce_1/NC catalyst under low-magnifications. (a)-(d) Low-magnification high-angle annular dark-field scanning transmission electron microscopy (HAADF-STEM) images of the Ce_1/NC catalyst. It was found that there were no particles in the Ce_1/NC catalyst from an overall observation.

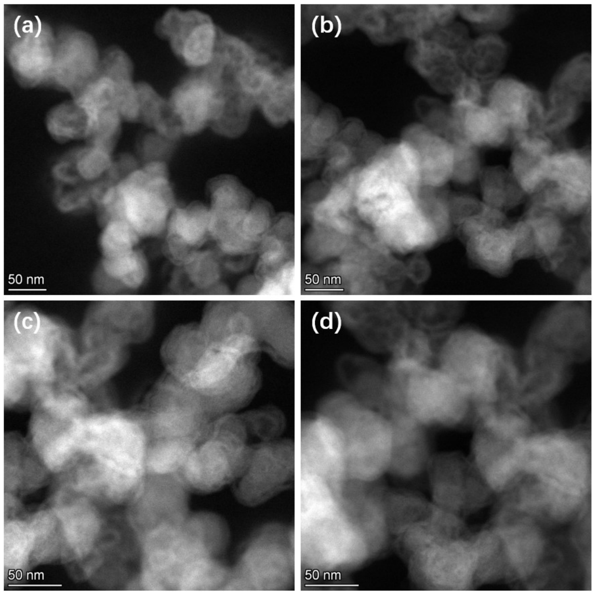

Figure S6. Microscopic investigation of the Ce_1/NC catalyst under medium-magnifications. (a)-(d) Medium-magnification high-angle annular dark-field scanning transmission electron microscopy (HAADF-STEM) images of the Ce_1/NC catalyst. It was identified that when enlarging different regions of the Ce_1/NC catalyst, no particles were observed.

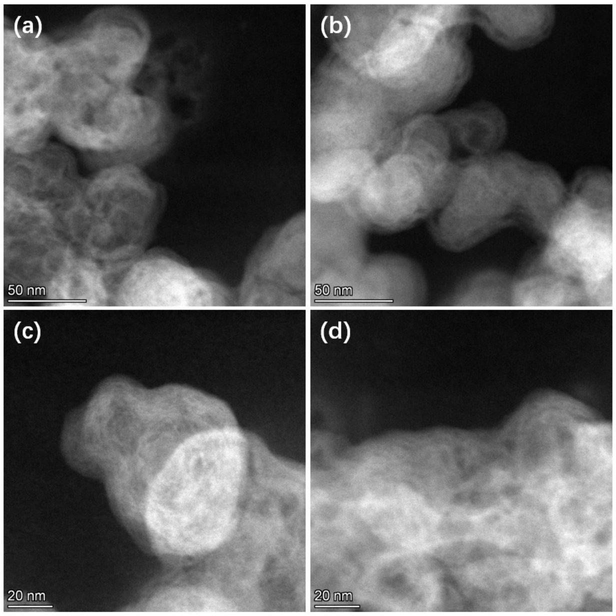

Figure S7. Microscopic exploration of the Ce_1/NC catalyst under high-magnifications. (a)-(d) High-magnification high-angle annular dark-field scanning transmission electron microscopy (HAADF-STEM) images of the Ce_1/NC catalyst. The existence of particles was further excluded on the Ce_1/NC catalyst by the high-magnification HAADF-STEM observation.

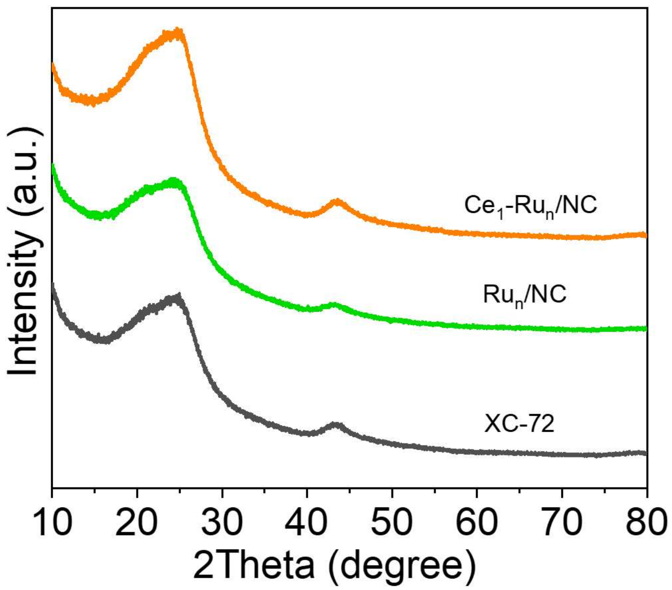

Figure S8. The XRD patterns of the Ru_n/NC, Ce_1-Ru_n/NC, and XC-72 support, respectively. It was unveiled that no Ru XRD peaks were found for the Ru_n/NC and Ce_1-Ru_n/NC catalysts except for the broad diffraction peaks of the XC-72 support, due to the ultrasmall Ru nanoclusters in them.

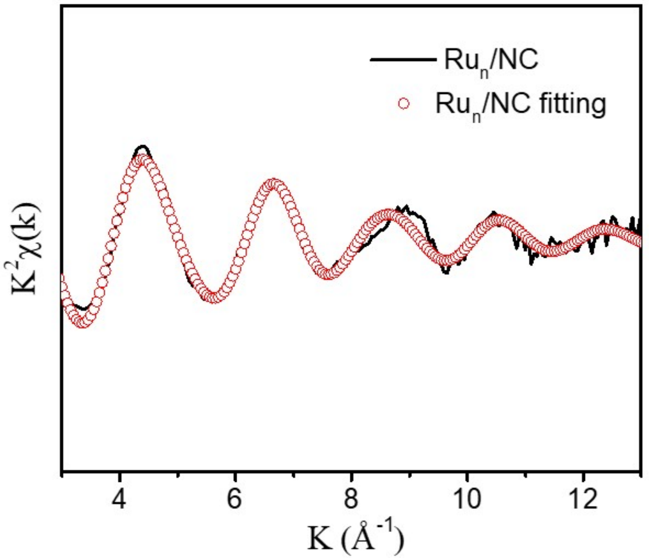

Figure S9. The Ru K-edge EXAFS fitting (red line) for the Ru_n/NC, shown in k^2 weighted *k*-space.

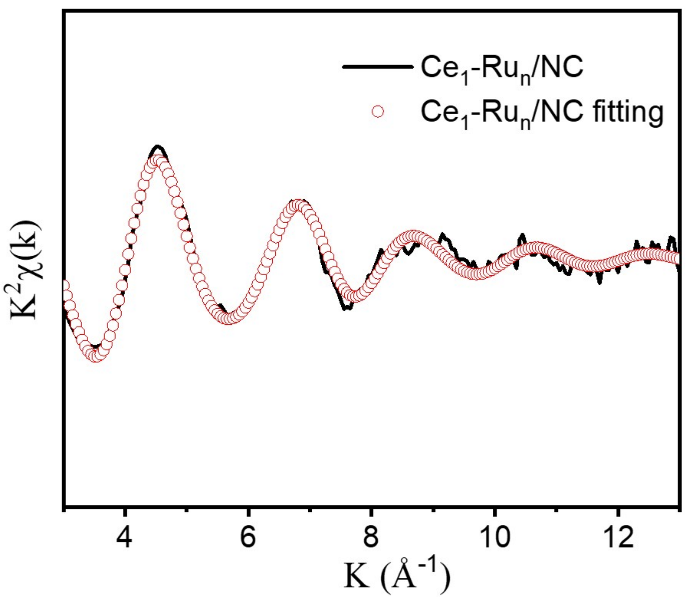

Figure S10. The Ru K-edge EXAFS fitting line for the Ce_1-Ru_n/NC (red line), shown in k^2 weighted *k*-space.

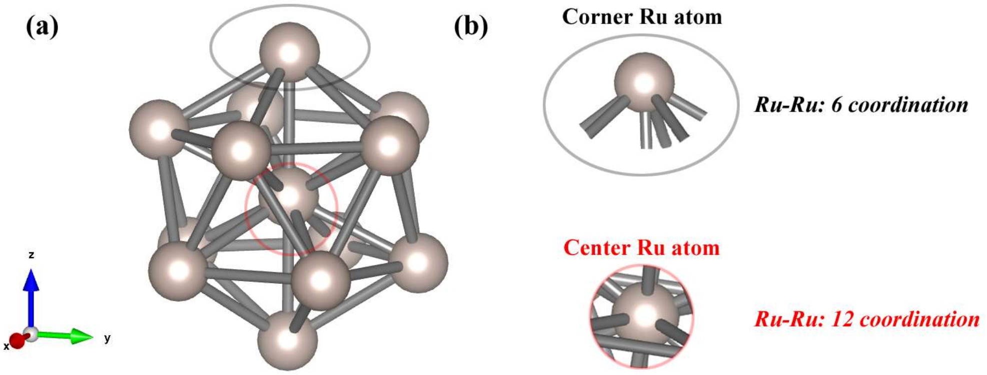

Figure S11. Structure model for the Ru_{13} nanocluster. (a) The simulated Ru_{13} nanocluster (corresponding to a Ru_n size of 1 nm). (b) The Ru-Ru coordination numbers of the corner and center Ru atoms in the Ru_{13} nanocluster. The Ru-Ru coordination numbers of the Ru_{13} nanocluster were calculated to be 6.5. Calculation details: the corner Ru atom numbers of the Ru_{13} nanocluster were 12 and each corner Ru atom possessed a Ru-Ru coordination number of 6. Thus, the total Ru-Ru coordination numbers of the corner Ru atoms were 12*6=72. By contrast, the center Ru atom of the Ru_{13} nanocluster displayed a Ru-Ru coordination number of 12, which made the total Ru-Ru coordination numbers of the Ru_{13} nanocluster as 72+12=84. As such, the average Ru-Ru coordination numbers of the Ru_{13} nanocluster would be 84/13=6.5.

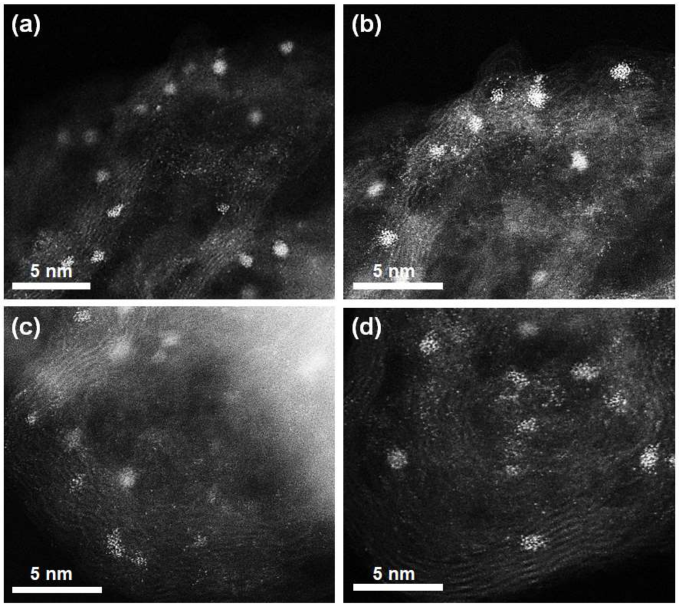

Figure S12. Microscopic measurements of the Ru_n/NC catalyst. (a)-(d) The aberration-corrected HAADF-STEM images of the Ru_n/NC catalyst. It was identified that Ru single atoms and small Ru_n nanoclusters coexisted on the NC support.

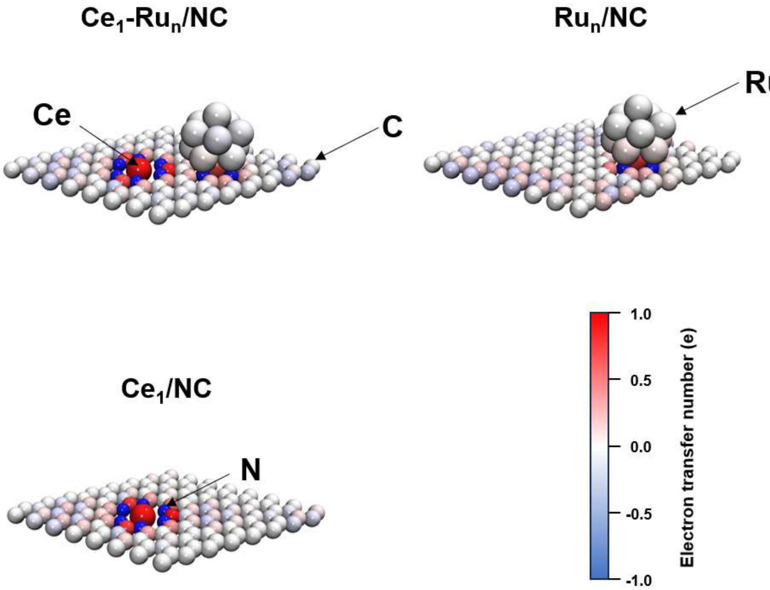

Figure S13. Colored Bader charge analysis for the Ce_1-Ru_n/NC, Ru_n/NC, and Ce_1/NC catalysts, respectively. As revealed by the Bader charge analysis, the electrons of Ce could be facilely transferred to Ru and the net electron transfer number from Ce single atom to Ru nanocluster was calculated to be 0.05. The red color region indicates electron loss and the blue color region indicates electron enriching.

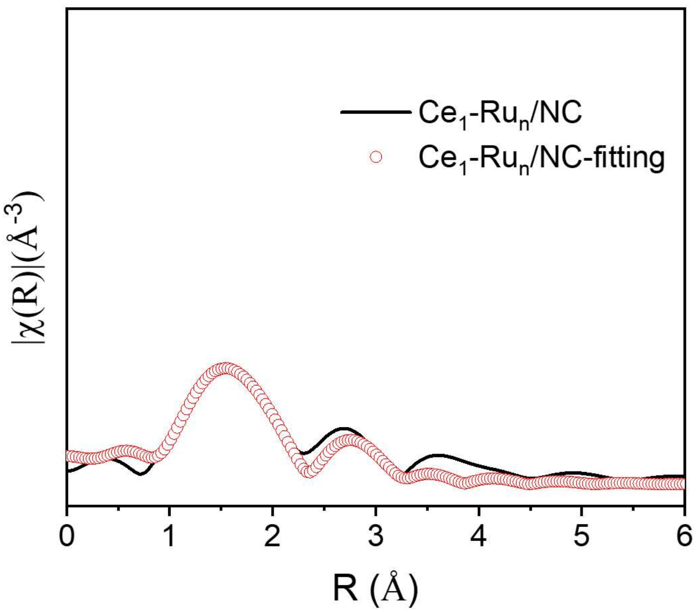

Figure S14. The Ce L_{III}-edge EXAFS fitting curve for the Ce_1-Ru_n/NC (red line), shown in k^2 weighted *R*-space.

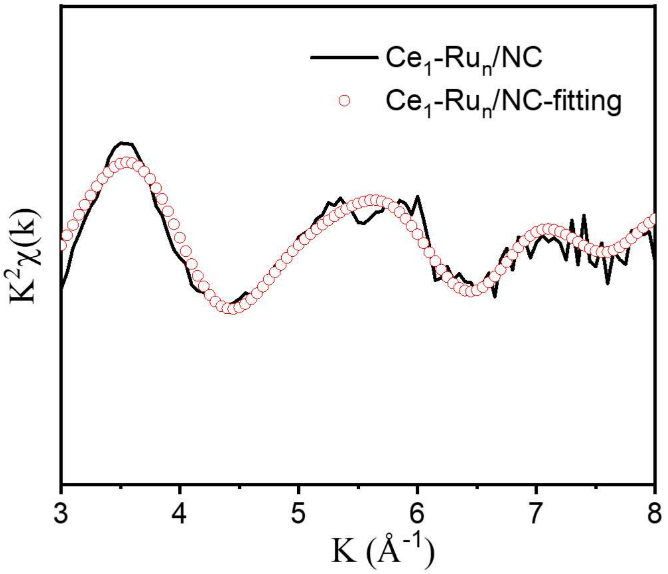

Figure S15. The Ce L_{III}-edge EXAFS fitting line for the Ce_1-Ru_n/NC (red line), shown in k^2 weighted *k*-space.

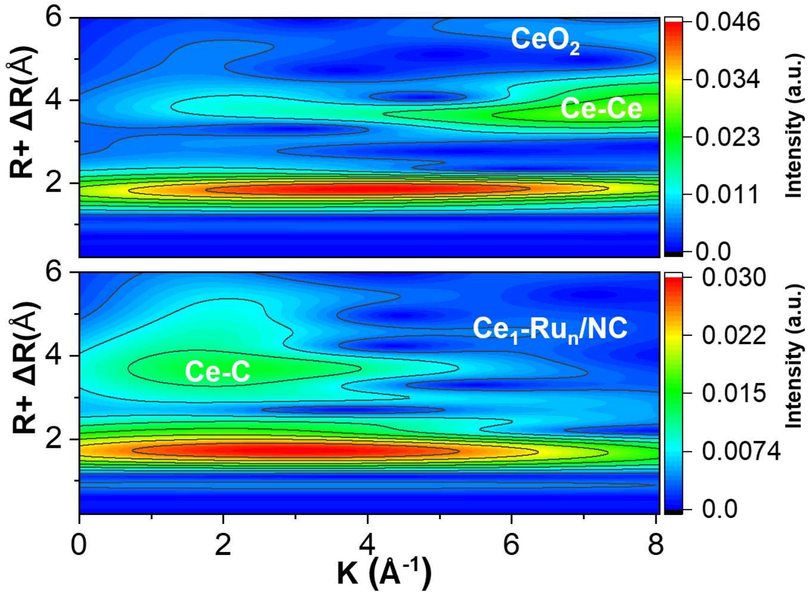

Figure S16. The wavelet transformation for the EXAFS signals of the Ce_1-Ru_n/NC catalyst and the reference CeO_2.

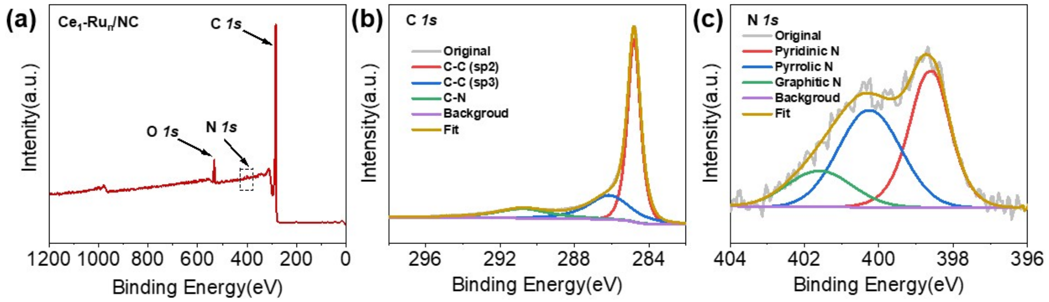

Figure S17. Spectroscopic measurements for the Ce_1-Ru_n/NC catalyst. (a) Survey XPS spectrum, (b) high-resolution C 1*s* XPS spectrum, (c) high-resolution N 1*s* spectrum of the Ce_1-Ru_n/NC catalyst.

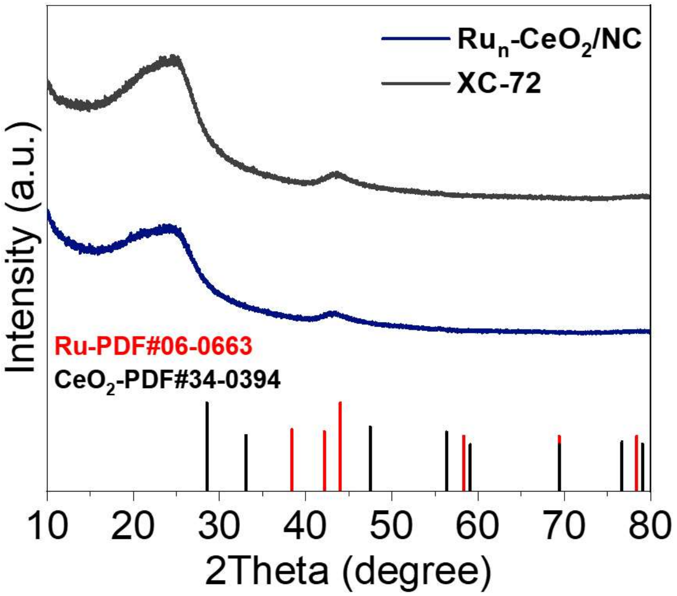

Figure S18. The XRD patterns of the Ru_n-CeO_2/NC catalyst and the XC-72 support. Wherein, no XRD peaks of Ru and CeO_2 were found for the Ru_n-CeO_2/NC catalyst due to the ultrafine particle sizes of them.

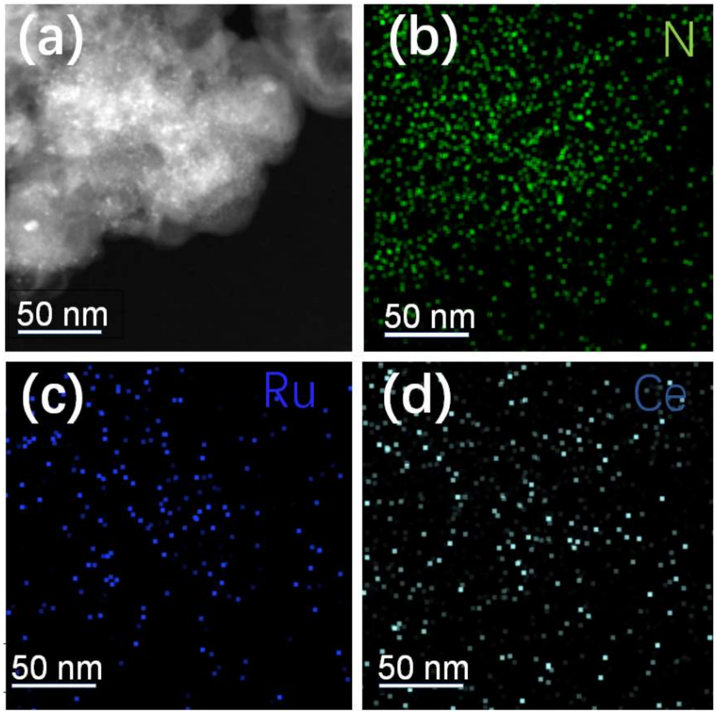

Figure S19. The overall EDS mapping analysis of the Ru_n-CeO_2/NC catalyst. (a) HAADF-STEM image and (b)-(d) corresponding EDS mapping images of the Ru_n-CeO_2/NC catalyst, which presented uniform dispersion of Ce and Ru element signals in the Ru_n-CeO_2/NC catalyst.

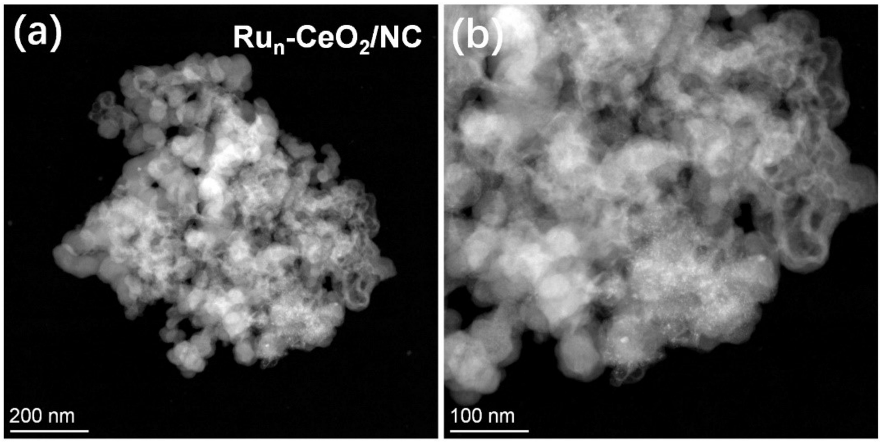

Figure S20. The HAADF-STEM observation of the Ru$_n$-CeO$_2$/NC catalyst. (a) and (b) HAADF-STEM images of the Ru$_n$-CeO$_2$/NC catalyst with varied magnifications. The Ru$_n$-CeO$_2$/NC was prepared by reducing the Ru cations impregnated CeO$_2$/NC. Since the hydrogen reduction was carried out at 250 °C, it was hardly to reduce the CeO$_2$ nanoparticles.

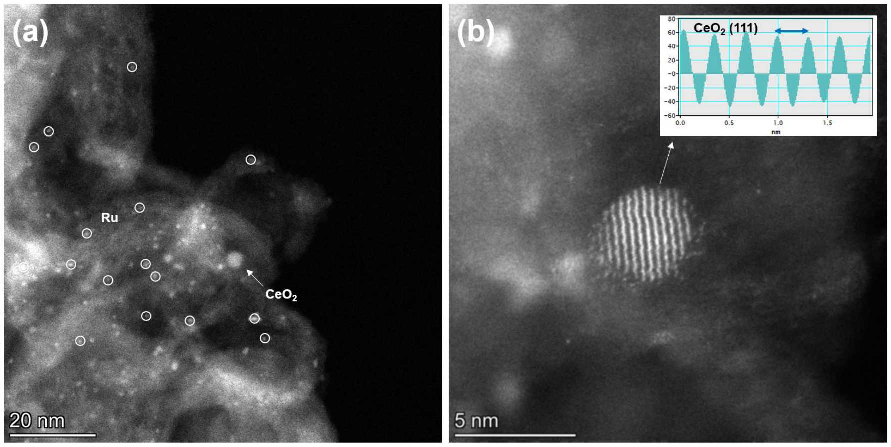

Figure S21. The aberration-corrected high-magnification HAADF-STEM observation of the Ru$_n$-CeO$_2$/NC catalyst. (a) The aberration-corrected HAADF-STEM image of the Ru$_n$-CeO$_2$/NC catalyst, which displayed the uniformly dispersed CeO$_2$ and Ru nanoparticles on it. (b) High-resolution HAADF-STEM image of a CeO$_2$ nanoparticle. It was identified that the lattice fringe of the relatively bigger particle was assigned to the (111) plane of CeO$_2$ (PDF no. 34-0394). The inset of (b) is the enlarged image of the CeO$_2$ particle.

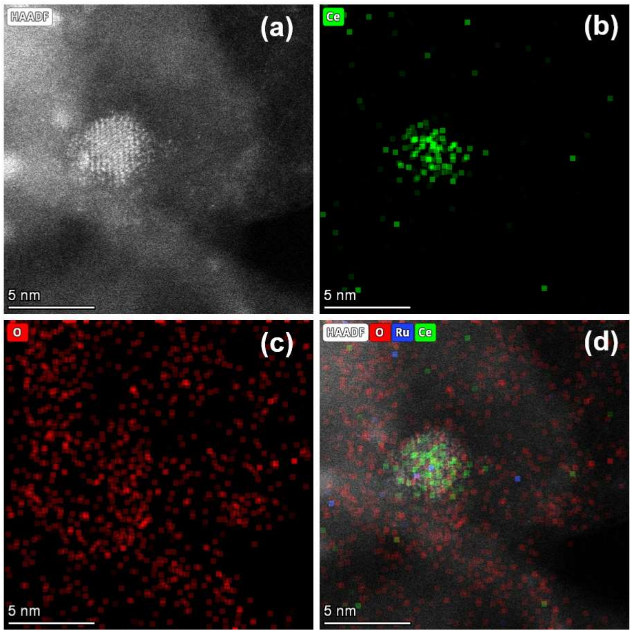

Figure S22. The localized EDS mapping analysis of the Ru_n-CeO_2/NC catalyst. (a) The aberration-corrected HAADF-STEM image and (b)-(d) corresponding EDS mapping images of the Ru_n-CeO_2/NC catalyst. Wherein, the relatively bigger particles in Ru_n-CeO_2/NC were confirmed to be the CeO_2.

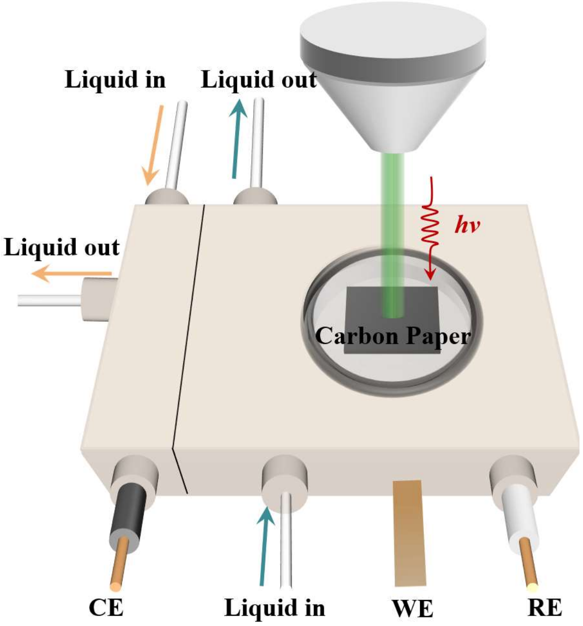

Figure S23. Schematic illustration of home-made electrochemical reaction cell for in situ Raman test using a standard three-electrode system in 1.0 M KOH electrolyte. The Ag/AgCl electrode was used as the reference electrode (RE), the platinum wire served as the counter electrode (CE) and the catalyst was loaded on the carbon paper (WE).

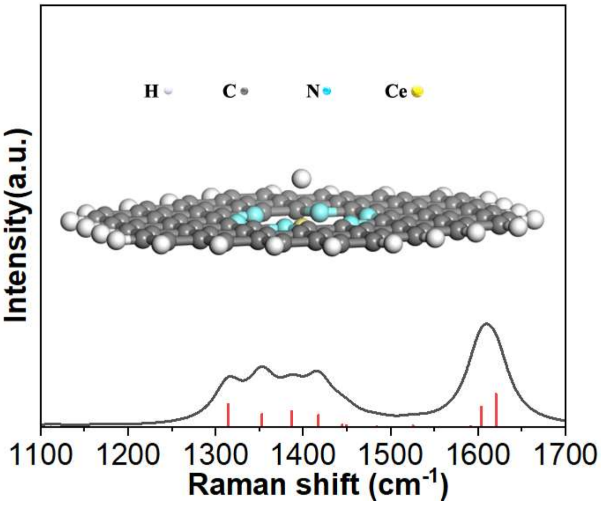

Figure S24. Theoretically fitted Raman curves of the Ce-N stretching vibration in Ce_1-Ru_n/NC in the presence of H species. Sharply different from the OH-promoting effect on the Ce-N stretching vibration, it was found that the H had a negligible impact on intensifying the Ce-N stretching vibration at 1533 cm^{-1} and 1390 cm^{-1}, respectively.

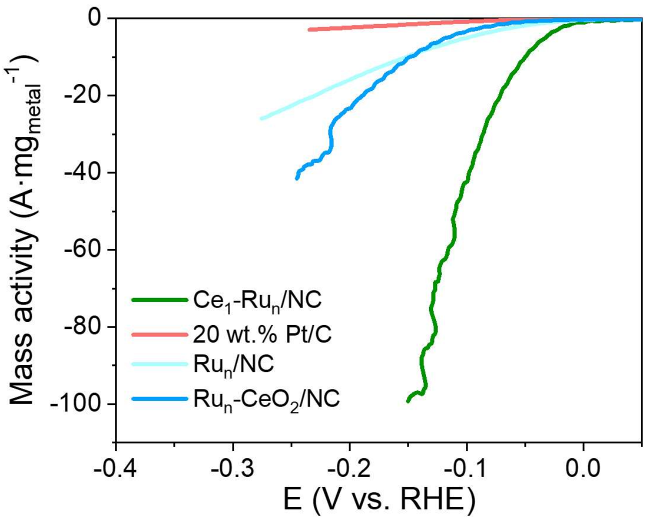

Figure S25. The mass activities of the Ce_1-Ru_n/NC, Ru_n/NC, Ru_n-CeO_2/NC and the commercial 20wt.% Pt/C catalyst. It was found that the mass activity of the Ce_1-Ru_n/NC was much larger than the commercial 20wt.% Pt/C. More importantly, the metal price of Ru (ca. 15 \$ g^{-1}, Sept 2023) was less than half of Pt (ca. 34 \$ g^{-1}, Sept 2023), making the Ce_1-Ru_n/NC catalyst particularly attractive for alkaline HER in terms of cost-effectiveness.

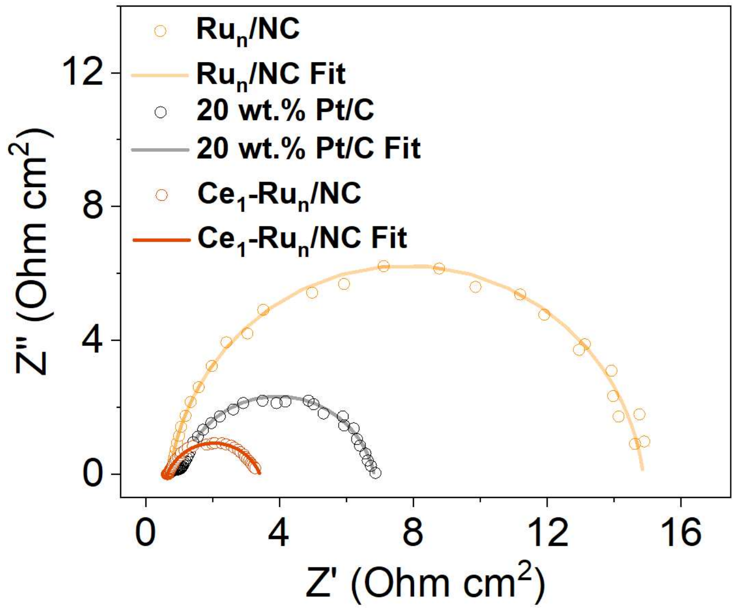

Figure S26. Nyquist curves for the Ce_1-Ru_n/NC, Ru_n/NC, and the commercial 20wt.% Pt/C, respectively. It was demonstrated that the charge transfer resistance (R_{ct}) of the Ce_1-Ru/NC was the lowest among these catalysts, revealing more rapid HER kinetics of the Ce_1-Ru_n/NC catalyst than other control catalysts.

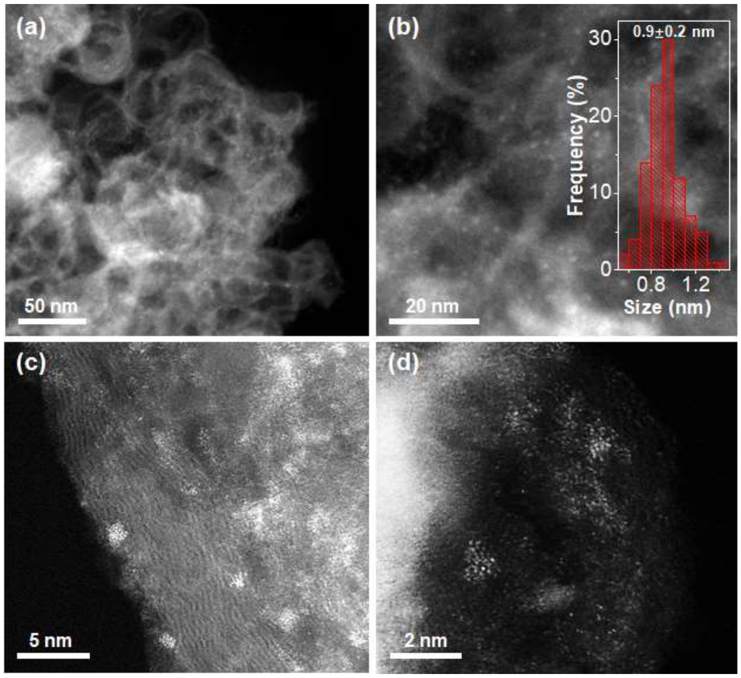

Figure S27. The microscopic measurements of the Ru_1-Ru_n/NC catalyst. (a) and (b) The HAADF-STEM images of the Ru_1-Ru_n/NC catalyst with varied magnifications. (c) and (d) The aberration-corrected HAADF-STEM images of the Ru_1-Ru_n/NC catalyst. The inset of (b) is the histogram of the particle-size distribution for Ru_n in the Ru_1-Ru_n/NC.

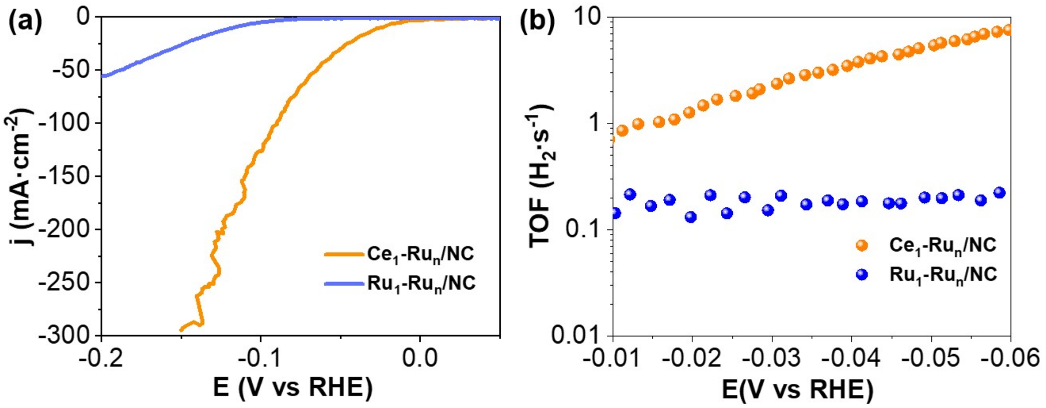

Figure S28. The alkaline hydrogen evolution examinations of the Ru_1-Ru_n/NC catalyst and the Ce_1-Ru_n/NC catalyst. (a) The LSV curves for the Ru_1-Ru_n/NC catalyst and the Ce_1-Ru_n/NC catalyst during the alkaline HER evaluations. (b) The TOF values of the Ru_1-Ru_n/NC catalyst and the Ce_1-Ru_n/NC catalyst measured in the potential range from -0.01 V to -0.06 V.

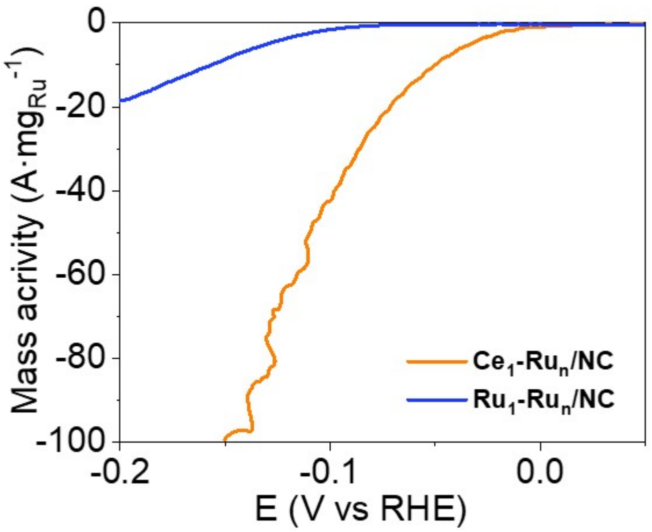

Figure S29. The mass activities normalized to per milligram Ru of the (a) Ce_1-Ru_n/NC catalyst and (b) Ru_1-Ru_n/NC catalyst.

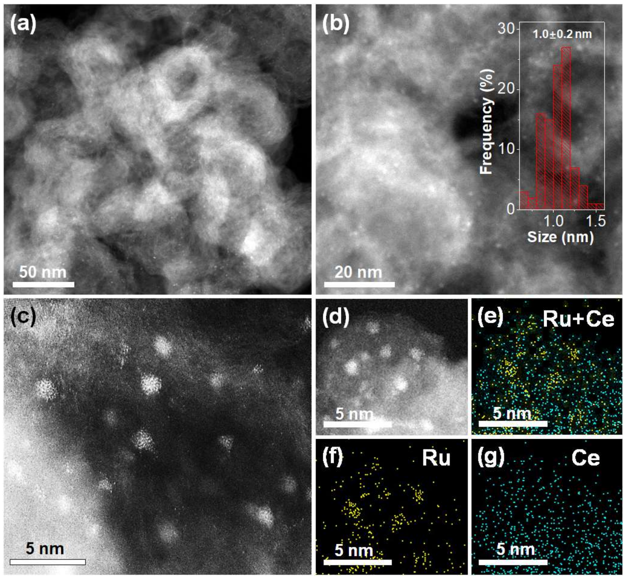

Figure S30. The microscopic characterizations of the post-reaction Ce_1-Ru_n/NC catalyst. (a) and (b) The HAADF-STEM images of the post-reaction Ce_1-Ru_n/NC catalyst. The inset of (b) is corresponding histogram of particle-size distribution of (b). (c) and (d): The aberration-corrected HAADF-STEM images of the post-reaction Ce_1-Ru_n/NC catalyst. (e)-(g) Corresponding energy-dispersive X-ray spectrometry (EDS) elementary mapping images of (d).

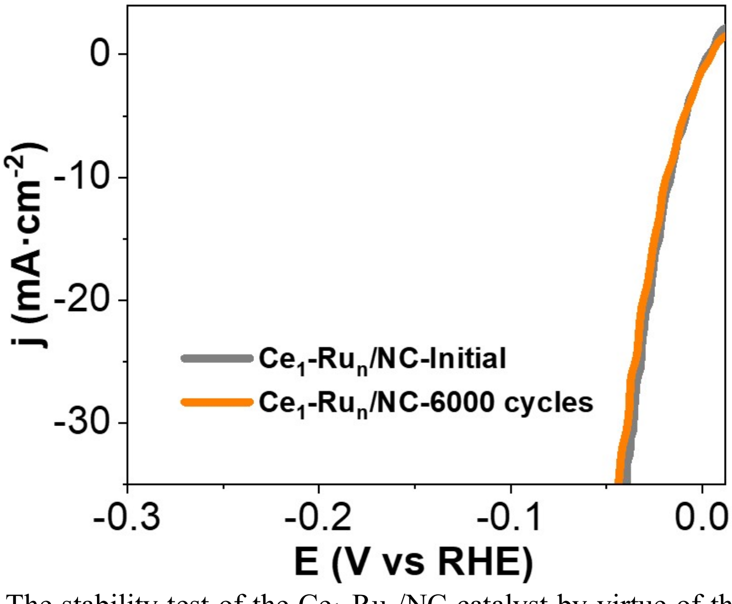

Figure S31. The stability test of the Ce_1-Ru_n/NC catalyst by virtue of the continuous cyclic voltammogram measurements conducted in 1.0 M KOH electrolyte.

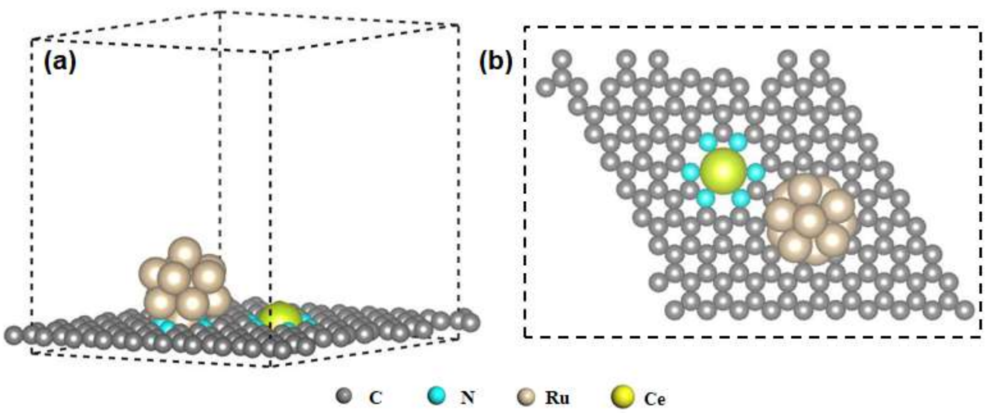

Figure S32. The structure model for the Ce_1-Ru_n/NC catalyst. (a) Side view and (b) top view of the dual Ce_1-Ru_{13} structural model for the simulation of the Ce_1-Ru_n/NC catalyst.

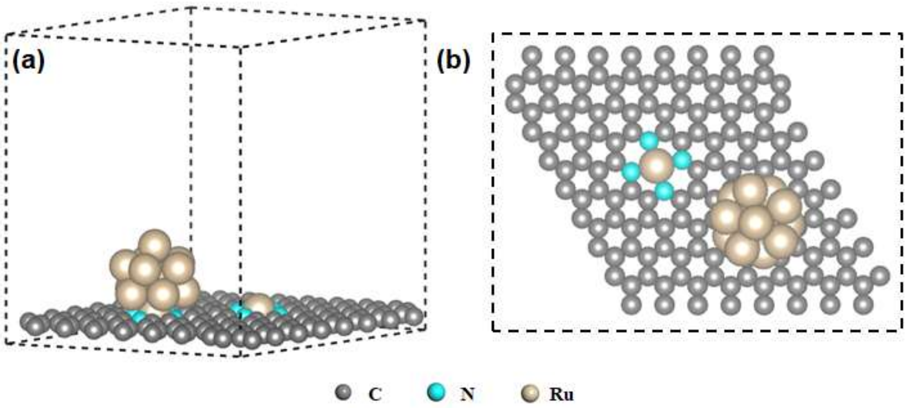

Figure S33. The structure model for the Ru_n/NC catalyst. (a) Side view and (b) top view of the dual Ru_1-Ru_{13} structural model for the simulation of the Ru_n/NC catalyst.

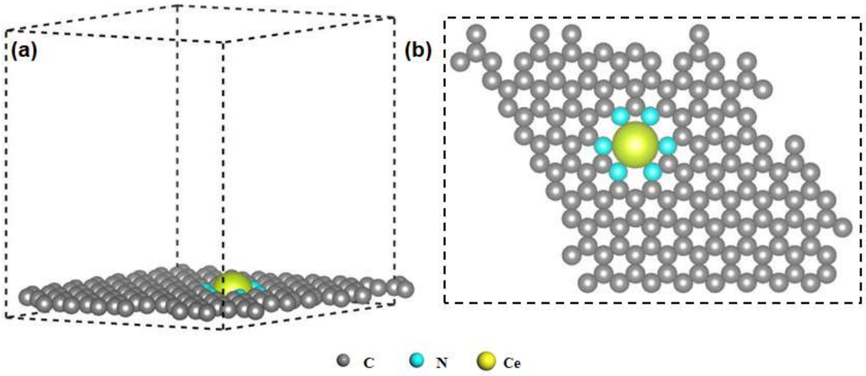

Figure S34. The structure model for the Ce_1/NC catalyst. (a) Side view and (b) top view of the Ce_1 structural model for the simulation of the Ce_1/NC catalyst.

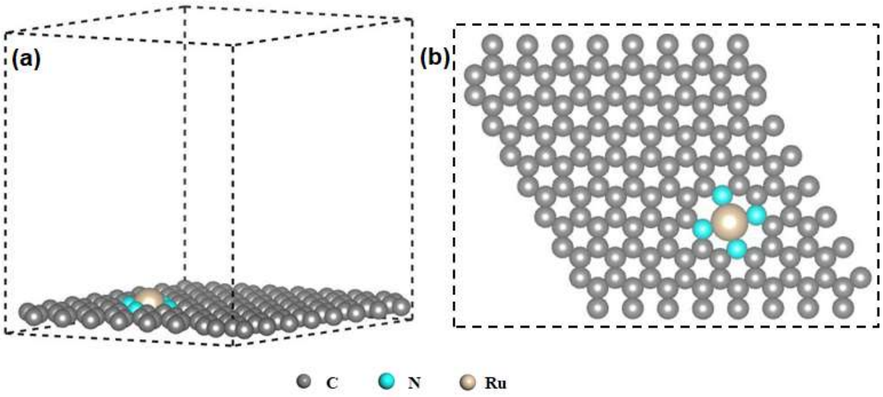

Figure S35. The structure model for the Ru1/NC catalyst. (a) Side view and (b) top view of the Ru1 structural model for the simulation of the Ru1/NC catalyst.

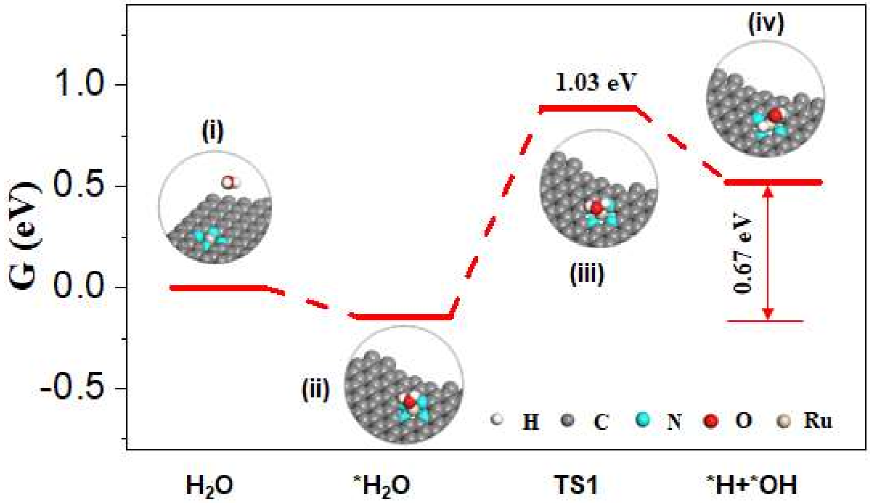

Figure S36. The Gibbs free energy diagrams for water dissociation over the Ru single atom catalyst (Ru1/NC). The insets of the picture are corresponding structure model for each step.

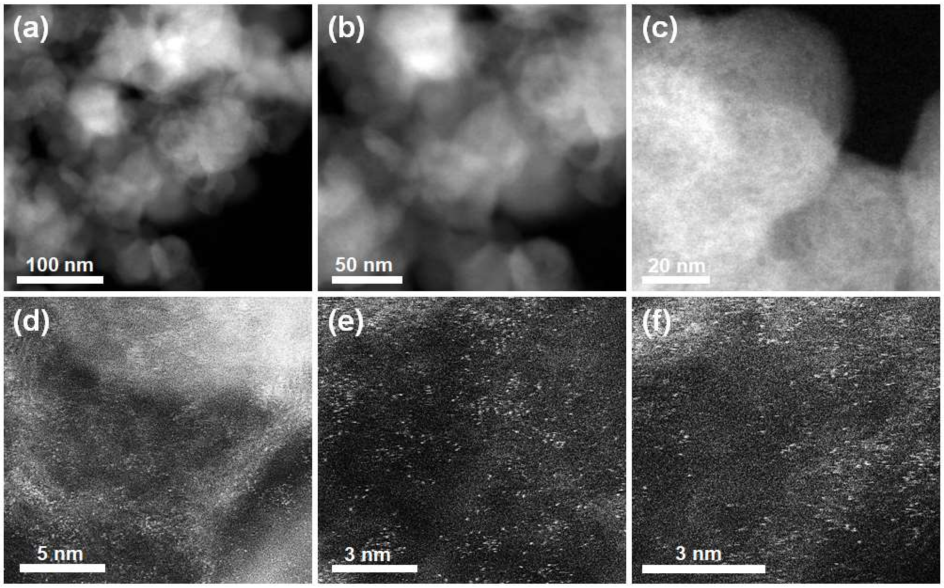

Figure S37. The microscopic measurements of the Ru_1/NC catalyst. (a)-(f) The aberration-corrected HAADF-STEM images of the Ru_1/NC catalyst with varied magnifications.

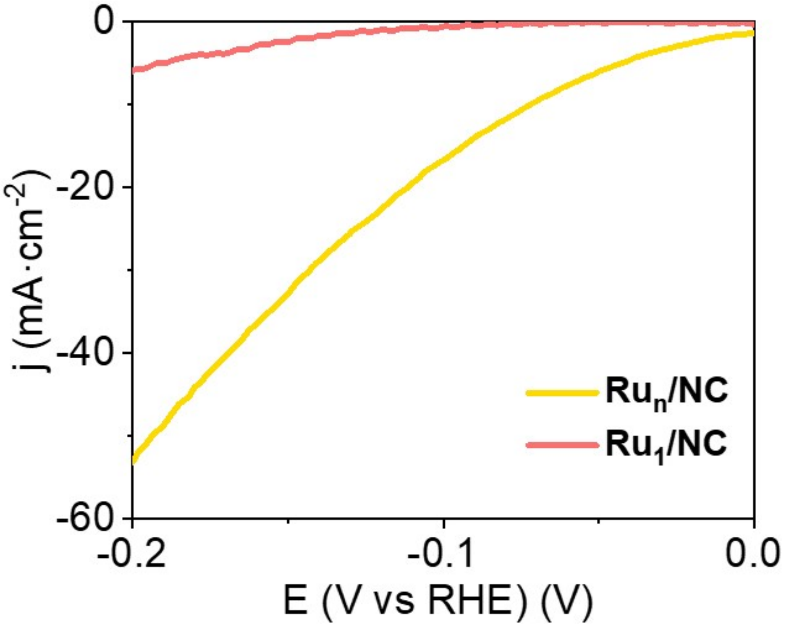

Figure S38. (a) The LSV curves for the Ru_1/NC catalyst and the Ru_n/NC catalyst during the alkaline HER evaluations. The Ru loading amounts of the Ru_1/NC catalyst and the Ru_n/NC catalyst were 0.2wt.% and 1.2wt.%, respectively.

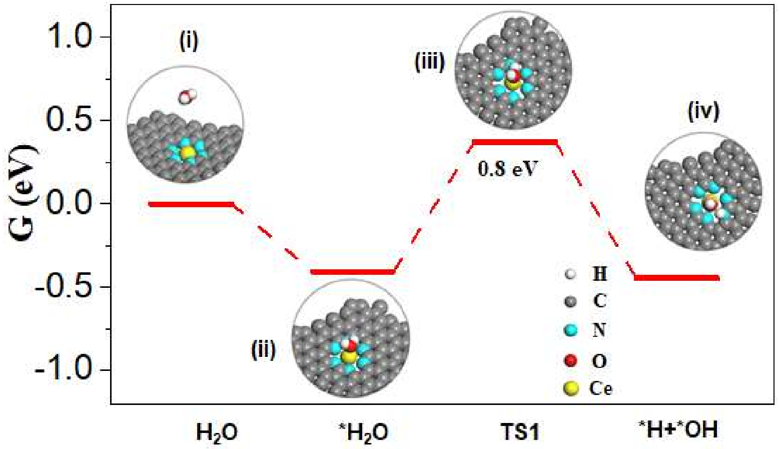

Figure S39. The Gibbs free energy diagrams for water dissociation over the Ce_1/NC catalyst. The insets of the picture are corresponding structure model for each step.

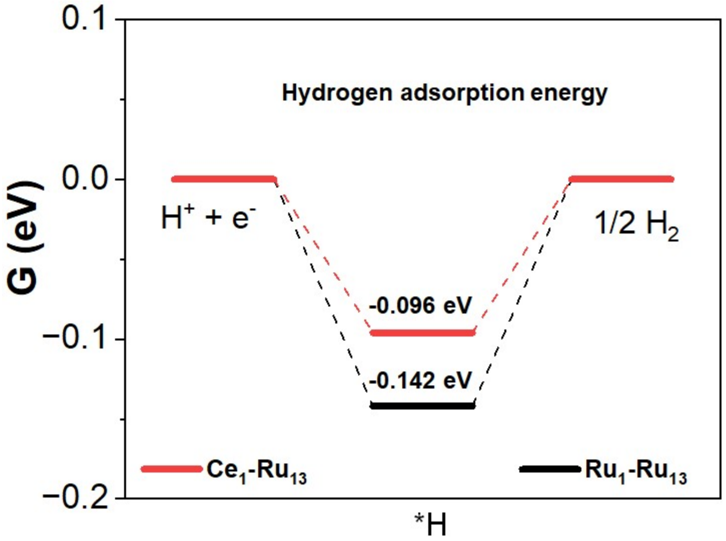

Figure S40. The H adsorption energies of the dual Ce_1-Ru_{13} site and the dual Ru_1-Ru_{13} site calculated by the CANDEL implicit solvation model method.

Supplementary Tables

Table S1. The metal loading amount of the main catalysts in current work as measured by the ICP-OES technique.

Catalyst	Ru loading (wt.%)	Ce loading (wt.%)
Ce_1/NC	------	0.03
Ru_n/NC	1.2	------
Ru_1-Ru_n/NC	1.1	------
Ce_1-Ru_n/NC	1.1	0.03

The metal loading was determined by the inductively coupled plasma optical emission spectrometer (ICP-OES) measurement.

Table S2. The best-fitted EXAFS results of Ru_n/NC and Ce_1-Ru_n/NC.

Sample	Shell	CN	R (Å)	σ^2 (10^{-2} Å^2)	ΔE_0 (eV)	r-factor (%)
**Ru foil**	Ru-Ru	12	2.67	0.4	4.1	1.6
**Ru_n/NC**	Ru-N	3.8	2.03	0.5	-6.2	1.3
	Ru-Ru	2.3	2.73	2.0	-6.2	
**Ce_1-Ru_n/NC**	Ru-N	4.3	2.03	0.8	-4.3	0.7
	Ru-Ru	2.1	2.67	2.2	-4.3	
**Ce_1-Ru_n/NC**	Ce-N	6.1	2.11	1.2	6.5	1.8

CN is the coordination number, R is the average distance for different coordination pairs, σ^2 is the Debye-Waller factor, and ΔE_0 is the inner potential correction. The accuracies of the above parameters are estimated as CN, ±20%; R, ±1%; σ^2, ± 20%; ΔE_0 , ±20%. The data range used for data fitting in k-space (Δk) and R-space (ΔR) are 3.0-13.0 Å^{-1} and 1.0-2.9 Å for Ru, 3.0-8.0 Å^{-1} and 1.0-2.0 Å for Ce, respectively.

Table S3. The loading amount of the Ru or Pt of the tested catalyst obtained by the ICP-OES measurement.

Catalyst	Ru loading (wt.%)	Pt loading (wt.%)
Ce_1-Ru_n/NC	1.1	------
Ru_n/NC	1.2	------
Ru_n-CeO_2/NC	1.3	------
Commercial Pt/C	------	20

The metal loading was determined by the inductively coupled plasma optical emission spectrometer (ICP-OES) measurement.

Table S4. The mass activity comparison between the Ce_1-Ru_n/NC catalyst and previously reported Ru-based catalyst at -0.05 V *vs.* RHE for alkaline HER performed in 1M KOH electrolyte.

Catalysts	Ru loading (wt.%)	Mass activity (A mg_{Ru}^{-1})	References
Ru/p-NC	0.7	ca. -30	2
Ru-NP@N-BP	0.44	-24	3
Ru–TA/ACC	0.058	ca. -18	4
Ru@Co-NC-800	0.35	-12	5
Ce_1-Ru_n/NC	1	-10.1	This work
Ru/WC	12.19	-9.33	6
RuBi SAA/Bi@OG	NA	-8.5	7
DR-Ru	0.8	-8	8
Ru-HPC	5.55	-7.8	9
Ru/OMSNNC	1	-7.5	10
Ru-ZIF-900	0.18	-6.4	11
Ru/Na^+, K^+-PC	3.1	-6.3	12
P-Ru/C	11.9	-5.2	13
Ru@Cu-TM	NA	-4.87	14
RuCo@RuSACoSA-NMC	1.43	-4.85	15
Ru NRs/TiN	2.26	-4.84	16
Ru/TiC	NA	-4.3	17
Ru/WNO@C	3.37	-4.2	18

CoRu-O/A@HNC-2	1.28	-4.1	19
β-Ni(OH)$_2$/Ni-Ru SAs	1.17	-4.08	20
Ru/Co@OG	6.9	-3.99	21
VO-Ru/HfO$_2$-OP	0.9	-3.5	22
Ru NPs/NC-900	10.5	-2.65	23
RuCo@NC-750	1.56	-2.6	24
Ru ADC	4.25	-2.55	25
Ru$_n$-Ru$_s$/NC	4.01	-2.45	26
RuGd-rGO	5.45	-2.3	27
Ru-CN/MC	5.21	-2.26	28
Ru$_{1,n}$-ZnFe$_2$O$_x$-C	8.3	-2.21	29
Ru-NMCNs-500	3.04	-2.1	30
MSOR$_1$	4.09	-2.07	31
Ru@CN-0.16	3.18	-1.99	32
NiCoRu$_{0.2}$/SP	0.9	-1.83	33
Ru/V$_2$O$_3$-CC	0.37	-1.63	34
Ru(3mL)-MC	2.65	-1.61	35
Ru@CQDs	3.78	-1.61	36
Ru/S-rGO	10.85	-1.58	37
CoSA-NC@Ru	0.32	-1.5	38
Ru/TiO$_2$-Vo@C-15	2.9	-1.29	39
Ru–MoS$_2$@PPy	6.05	-1.27	40

Ru/NiFe LDH-F/NF	10	-1.24	41
Ru-CON 800 ºC	13.03	-1.2	42
CC@$MoSe_2$/Ru	0.78	-1.14	43
Ru/NC-400	6	-1.1	44
Ru/TNTA	NA	-1.1	45
Ni@Ru/CNS	6.78	-0.95	46
RMC-500	7.13	-0.88	47
HP-Ru/C	3.4	-0.86	48
Ru/MoO_2	21.26	-0.85	49
Ru/NC-0.01	16.7	-0.83	50
Ru–WSe_2	4.25	-0.8	51
Ru/NSC-200	2.58	-0.8	52
SL-RuO_2/C	21.66	-0.78	53
RuNi NSs	NA	-0.74	54
NC@Ru_{sa}-CoP	13.87	-0.74	55
N-RuP/NPC	10.1	-0.73	56
Ru/ZC	4	-0.71	57
Ru@F–Ni_3N	0.11	-0.7	58
Ru/Ni/WC@NPC	4.13	-0.6	59
Ru/CoO	NA	-0.6	60
Ru@NCN	9.1	-0.58	61
S-RuP_2/NPC	4.8	-0.58	62

Ru MIs-MoS_2	10	-0.55	63
3.0 wt%Ru/rGO	3	-0.5	64
G(Zn)-Ru(100:1)	13.5	-0.46	65
Ru/B–Ni_2P/Ni_5P_4	6.28	-0.43	66
RuO_x@TiO_2	10	-0.43	67
Ru/H–S,N–C	17.1	-0.42	68
Ru/α-MoC	7.65	-0.42	69
$CoRu_{0.05}$@Gr	2.48	-0.42	70
Ru@RuP/PC-2	14.9	-0.41	71
Ru/AgCl@Ag-20%	NA	ca. -0.4	72
CNT-V-Fe-Ru	17.84	-0.4	73
Ru-N/DOMMC	2.6	-0.31	74
Co@RuCo-3	4.31	-0.3	75
RuPx@NPC	12.64	-0.3	76
Ru-CoO@SNG	ca. 9.9	-0.29	77
Ru-HCRLs850	23.5	-0.22	78
RuCo@NG/N-GNs	64	-0.21	79
Ru_{NP}-Ru_{SA}@CFN-800	12	-0.21	80
RuO_2@MoS_2	NA	-0.21	81
Ru-HMT-MP-7	0.27	-0.2	82
Ru–Cl–N SAC	24.3	-0.2	83
RuNi-0	NA	-0.2	84

Ru@SC-CDs2:10	21.6	-0.2	85
np-$Cu_{53}Ru_{47}$	56.53	-0.19	86
Ru-NiCo-LDH	NA	-0.19	87
Ru/Co_4N-CoF_2	5	-0.17	88
RuAu-0.2	74	-0.16	89
$RuSe_2$	39	-0.15	90
RuP_2@PC	14.6	-0.14	91
Ru/3DNCN	29	ca. -0.14	92
Ni_5P_2-mPtRu/NF	18.6	-0.12	93
Ru_1Ni_1-NCNFs	28.2	-0.12	94
Sr_2RuO_4	29.6	-0.1	95
Ru@ZIF-L(Co)/FL-$Ti_3C_2T_x$	~25	ca. -0.1	96
Ru-Cr_2O_3/NG	17	-0.08	97
$Ru_{1.0}$/NF	1.1	ca. -0.07	98
$CoMoRu_{0.25}O_x$/NF	1.86	-0.04	99
$Cu_{1.94}S$@Ru	55	-0.04	100
RuV–NiCoP/NF	NA	-0.03	101
CF@Ru–CoCH	NA	-0.03	102
$Ru(OH)_x$/Ag	3.27	-0.01	103
W-Ru/NiP_2	NA	-0.01	104

Table S5. The mass activity comparison between the Ce_1-Ru_n/NC catalyst and previously reported Ru-based catalyst at -0.1 V *vs*. RHE for alkaline HER performed in 1M KOH electrolyte.

**Catalysts**	**Ru loading (wt.%)**	**Mass activity (A mg_{Ru}^{-1})**	**References**
Ru-NP@N-BP	0.44	ca. -50	3
Ru–TA/ACC	0.058	ca. -45	4
Ru/p-NC	0.7	NA	2
Ru@Co-NC-800	0.35	NA	5
Ce_1-Ru_n/NC	1	-44.3	This work
DR-Ru	0.8	-40	8
RuBi SAA/Bi@OG	NA	-27.3	7
Ru/WC	12.19	-24.83	6
β-$Ni(OH)_2$/Ni-Ru SAs	1.17	-23.9	20
Ru@WNO-C	0.9	-22.7	105
PtCoRu NAs	0.85	-21.1	106
Ru-ZIF-900	0.18	-19.8	11
Ru-HPC	5.55	-17.9	9
Ru/Na^+, K^+-PC	3.1	-15.7	12
RuCo@RuSACoSA-NMC	1.43	-14.9	15
Ru_1/D-NiFe LDH	1.2	-14.7	107
Ru/OMSNNC	1	-14.7	10
P-Ru/C	11.9	-12.9	13

VO-Ru/HfO_2-OP	0.9	-12	22
CoRu-O/A@HNC-2	1.28	-10.1	19
Ru/TiC	NA	-9.9	17
Ru_n-Ru_s/NC	4.01	-8.8	26
CC@$MoSe_2$/Ru	0.78	-7.81	43
Ru/TNTA	NA	-7.1	45
$NiCoRu_{0.2}$/SP	0.9	-7	33
RuGd-rGO	5.45	-5.98	27
Ru NPs/NC-900	10.5	-5.8	23
RuCo@NC-750	1.56	-5.8	24
Ru-NMCNs-500	3.04	-5.2	30
Ru ADC	4.25	-4.89	25
Ru/NiFe LDH-F/NF	10	-4.79	41
S-RuP@NPSC-900	0.8	-4.7	108
Ru@CN-0.16	3.18	-4.52	32
Ru/S-rGO	10.85	-3.87	37
(Ru) RP-CPM	13.7	-3.85	109
Ru/V_2O_3-CC	0.37	-3.49	34
Ru/NC-400	6	-3.4	44
Ru/TiO_2-Vo@C-15	2.9	-3.17	[110]
NCPO-Ru NCs	3.47	-3.02	[111]
Ru–MoS_2@PPy	6.05	-2.87	40

Ru@CQDs	3.78	-2.82	36
Ru/Mo_2CT_x	1.2	-2.8	[112]
N-RuP/NPC	10.1	-2.72	56
Ru NCs/VN-C_3N_4	5.57	-2.69	[113]
RuP_x@NPC	12.64	-2.68	76
Ru@F–Ni_3N	0.11	-2.46	58
Ni@Ru/CNS	6.78	-2.42	46
Ir−Ru DSACs	NA	-2.36	[114]
HP-Ru/C	3.4	-2.3	48
Ru/NSC-200	2.58	-2.22	52
NC@Ru_{sa}-CoP	13.87	-2.21	55
Ru/CoO	NA	-2.19	60
Ru/NC-0.01	16.7	-2.15	50
CNT-V-Fe-Ru	17.84	-2.07	73
Ru–WSe_2	4.25	-1.9	51
RMC-500	7.13	-1.89	47
Ru/MoO_2	21.26	-1.88	49
Ru/ZC	4	-1.88	57
Ru@NCN	9.1	-1.83	61
Co@RuCo-3	4.31	-1.82	75
RuO_x@TiO_2	10	-1.81	67
PdO−RuO_2/C	10.7	-1.7	[115]

S-RuP_2/NPC	4.8	-1.63	62
3.0 wt%Ru/rGO	3	-1.46	64
Ru/B–Ni_2P/Ni_5P_4	6.28	-1.46	66
Ru MIs-MoS_2	10	-1.45	63
G(Zn)-Ru(100:1)	13.5	-1.43	65
Ru/α-MoC	7.65	-1.42	69
$CoRu_{0.05}$@Gr	2.48	-1.35	70
Ru/Ni/WC@NPC	4.13	-1.29	59
Ru/WNO@C	3.37	-1.22	18
Ru@RuP/PC-2	14.9	-1.01	71
Ru-CoO@SNG	ca. 9.9	-0.94	77
RuP_2@PC	14.6	-0.93	91
Ru-N/DOMMC	2.6	-0.85	74
$RuSe_2$	39	-0.82	90
Ru-HMT-MP-7	0.27	-0.79	82
RuNi-0	NA	-0.62	84
RuO_2@MoS_2	NA	-0.52	81
Ni_5P_2-mPtRu/NF	18.6	-0.51	93
Ru–Cl–N SAC	24.3	-0.5	83
np-$Cu_{53}Ru_{47}$	56.53	-0.4	86
Ru@SC-CDs2:10	21.6	-0.39	85
RuCo@NG/N-GNs	64	-0.39	79

Sr_2RuO_4	29.6	-0.37	95
$CoMoRu_{0.25}O_x$/NF	1.86	-0.37	99
Ru-Cr_2O_3/NG	17	-0.36	97
Ru/N,S- Go	7.2	-0.35	[116]
Ru/3DNCN	29	ca. -0.31	92
Cu–RuS_2/Ru	30.2	-0.29	[117]
Ru@ZIF-L(Co)/FL-$Ti_3C_2T_x$	~25	ca. -0.28	96
Ru/Co_4N-CoF_2	5	-0.28	88
CF@Ru–CoCH	NA	-0.16	102
$Cu_{1.94}S$@Ru	55	-0.11	100
$Ru(OH)_x$/Ag	3.27	-0.05	103
W-Ru/NiP_2	NA	-0.02	104

Supplementary reference:

1 Chen Y, *et al.* Isolated Single Iron Atoms Anchored on N-Doped Porous Carbon as an Efficient Electrocatalyst for the Oxygen Reduction Reaction. *Angew. Chem. Int. Ed.* **56**, 6937-6941 (2017).

2 Li Y, *et al.* Ru single atoms and nanoclusters on highly porous N-doped carbon as a hydrogen evolution catalyst in alkaline solutions with ultrahigh mass activity and turnover frequency. *Journal of Materials Chemistry A.* **9**, 12196-12202 (2021).

3 Zhi Q, *et al.* Ultralow loading of ruthenium nanoparticles on nitrogen-doped porous carbon enables ultrahigh mass activity for the hydrogen evolution reaction in alkaline media. *Catalysis Science & Technology.* **11**, 3182-3188 (2021).

4 Chen J, Wang H, Gong Y, Wang Y. Directly immobilizing a Ru–tannic acid linkage coordination complex on carbon cloth: an efficient and ultrastable catalyst for the hydrogen evolution reaction. *Journal of Materials Chemistry A.* **7**, 11038-11043 (2019).

5 Gao H, *et al.* Ruthenium and cobalt bimetal encapsulated in nitrogen-doped carbon material derived of ZIF-67 as enhanced hydrogen evolution electrocatalyst. *Applied Surface Science.* **494**, 101-110 (2019).

6 Wang C, *et al.* High-temperature shock enabled synthesis of ultrafine Ru nanoparticles anchoring onto tungsten carbide with strong metal-support interaction for ampere-level current density hydrogen evolution. *Journal of Alloys and Compounds.* **967**, 171667 (2023).

7 Zhao X, *et al.* A Double Atomic-Tuned RuBi SAA/Bi@OG Nanostructure with Optimum Charge Redistribution for Efficient Hydrogen Evolution. *Angewandte Chemie International Edition.* **62**, e202300879 (2023).

8 Li Y, *et al.* High mass-specific reactivity of a defect-enriched Ru electrocatalyst for hydrogen evolution in harsh alkaline and acidic media. *Science China Materials.* **64**, 2467-2476 (2021).

9 Qiu T, *et al.* Highly exposed ruthenium-based electrocatalysts from bimetallic metal-organic frameworks for overall water splitting. *Nano Energy.* **58**, 1-10 (2019).

10 Wu Y-L, *et al.* Ordered Macroporous Superstructure of Nitrogen-Doped Nanoporous Carbon Implanted with Ultrafine Ru Nanoclusters for Efficient pH-Universal Hydrogen Evolution Reaction. *Advanced Materials.* **33**, 2006965 (2021).

11 Xing L, *et al.* Atomically dispersed ruthenium sites on whisker-like secondary microstructure of porous carbon host toward highly efficient hydrogen evolution. *Journal of Materials Chemistry A.* **8**, 3203-3210 (2020).

12 Duan M, *et al.* Boosting alkaline hydrogen evolution performance by constructing ultrasmall Ru clusters/Na+, K+-decorated porous carbon composites. *Nano Research.* **16**, 8836-8844 (2023).

13 Zhao Y, Wang X, Cheng G, Luo W. Phosphorus-Induced Activation of Ruthenium for Boosting Hydrogen Oxidation and Evolution Electrocatalysis. *ACS Catalysis.* **10**, 11751-11757 (2020).

14 Han L, *et al.* The splanchnic mesenchyme is the tissue of origin for pancreatic fibroblasts during homeostasis and tumorigenesis. *Nature Communications.* **14**, 1 (2023).

15 Wang X, *et al.* Double-Tuned RuCo Dual Metal Single Atoms and Nanoalloy with Synchronously Expedited Volmer/Tafel Kinetics for Effective and Ultrastable Ampere-

Level Current Density Hydrogen Production. *Advanced Functional Materials.* **33**, 2301804 (2023).

16 Yang Y*, et al.* Engineering the strong metal support interaction of titanium nitride and ruthenium nanorods for effective hydrogen evolution reaction. *Applied Catalysis B: Environmental.* **317**, 121796 (2022).

17 Kim J, Jung S-M, Kim K-S, You S-H, Lee B-J, Kim Y-T. Highly Active Electrocatalyst based on Ultra-low Loading of Ruthenium Supported on Titanium Carbide for Alkaline Hydrogen Evolution Reaction. *J. Electrochem. Sci. Technol.* **13**, 417-423 (2022).

18 Zhang L-N*, et al.* Cable-like Ru/WNO@C nanowires for simultaneous high-efficiency hydrogen evolution and low-energy consumption chlor-alkali electrolysis. *Energy & Environmental Science.* **12**, 2569-2580 (2019).

19 Li G, Zheng K, Li W, He Y, Xu C. Ultralow Ru-Induced Bimetal Electrocatalysts with a Ru-Enriched and Mixed-Valence Surface Anchored on a Hollow Carbon Matrix for Oxygen Reduction and Water Splitting. *ACS Applied Materials & Interfaces.* **12**, 51437-51447 (2020).

20 Yan P*, et al.* "One Stone Five Birds" Plasma Activation Strategy Synergistic with Ru Single Atoms Doping Boosting the Hydrogen Evolution Performance of Metal Hydroxide. *Advanced Functional Materials.* **33**, 2301343 (2023).

21 Su P*, et al.* Exceptional Electrochemical HER Performance with Enhanced Electron Transfer between Ru Nanoparticles and Single Atoms Dispersed on a Carbon Substrate. *Angewandte Chemie International Edition.* **60**, 16044-16050 (2021).

22 Manigrasso J*, et al.* Author Correction: Visualizing group II intron dynamics between the first and second steps of splicing. *Nature Communications.* **13**, 1 (2022).

23 Jiang Z*, et al.* Lattice Strain and Schottky Junction Dual Regulation Boosts Ultrafine Ruthenium Nanoparticles Anchored on a N-Modified Carbon Catalyst for H2 Production. *Journal of the American Chemical Society.* **144**, 19619-19626 (2022).

24 Qu Y*, et al.* Thermal Emitting Strategy to Synthesize Atomically Dispersed Pt Metal Sites from Bulk Pt Metal. *J. Am. Chem. Soc.* **141**, 4505-4509 (2019).

25 Cao D, Wang J, Xu H, Cheng D. Construction of Dual-Site Atomically Dispersed Electrocatalysts with Ru-C5 Single Atoms and Ru-O4 Nanoclusters for Accelerated Alkali Hydrogen Evolution. *Small.* **17**, 2101163 (2021).

26 Yang C*, et al.* Electronic Structure-Dependent Water-Dissociation Pathways of Ruthenium-Based Catalysts in Alkaline H2-Evolution. *Small.* **19**, 2206949 (2023).

27 Zhao H*, et al.* Rapid and large-scale synthesis of ultra-small immiscible alloy supported catalysts. *Applied Catalysis B: Environmental.* **304**, 120916 (2022).

28 Liu X, Jin TL, Hood ZD, Tian C, Guo Y, Zhan W. Mechanochemically assisted synthesis of ruthenium clusters embedded in mesoporous carbon for an efficient hydrogen evolution reaction. *ChemElectroChem.* **6**, 2719-2725 (2019).

29 Qian C*, et al.* Competitive Coordination-Pairing between Ru Clusters and Single-Atoms for Efficient Hydrogen Evolution Reaction in Alkaline Seawater. *Small.* **18**, 2204155 (2022).

30 Peng J, Chen Y, Wang K, Tang Z, Chen S. High-performance Ru-based electrocatalyst composed of Ru nanoparticles and Ru single atoms for hydrogen evolution reaction in alkaline solution. *International Journal of Hydrogen Energy.* **45**, 18840-18849 (2020).

31 Zhang Y, Yang T, Li J, Zhang Q, Li B, Gao M. Construction of Ru, O Co-Doping MoS2 for

Hydrogen Evolution Reaction Electrocatalyst and Surface-Enhanced Raman Scattering Substrate: High-Performance, Recyclable, and Durability Improvement. *Advanced Functional Materials.* **33**, 2210939 (2023).
32 Wang J, Wei Z, Mao S, Li H, Wang Y. Highly uniform Ru nanoparticles over N-doped carbon: pH and temperature-universal hydrogen release from water reduction. *Energy & Environmental Science.* **11**, 800-806 (2018).
33 Li L*, et al.* Atomic ruthenium modification of nickel-cobalt alloy for enhanced alkaline hydrogen evolution. *Applied Catalysis B: Environmental.* **331**, 122710 (2023).
34 Fan X-Z, Pang Q-Q, Fan F, Yao H-C, Li Z-J. Ultra-fine Ru nanoparticles decorated V2O3 as a pH-universal electrocatalyst for efficient hydrogen evolution reaction. *International Journal of Hydrogen Energy.* **48**, 20577-20587 (2023).
35 Jiang Y*, et al.* Highly Efficient Oxygen-Modulated Ru-Based HER Electrocatalyst in a Wide pH Range. *ChemElectroChem.* **9**, e202101580 (2022).
36 Li W*, et al.* Carbon-Quantum-Dots-Loaded Ruthenium Nanoparticles as an Efficient Electrocatalyst for Hydrogen Production in Alkaline Media. *Advanced Materials.* **30**, 1800676 (2018).
37 Sun X*, et al.* Ultrasmall Ru Nanoparticles Highly Dispersed on Sulfur-Doped Graphene for HER with High Electrocatalytic Performance. *ACS Applied Materials & Interfaces.* **12**, 48591-48597 (2020).
38 Jeong Y-B, Hoon Ahn S. Selective surface deposition of trace amount of ruthenium onto a freestanding, fibrous carbon monolith for pH-universal hydrogen evolution reaction. *Chemical Engineering Journal.* **437**, 135322 (2022).
39 Wei Z*, et al.* Oxygen-deficient TiO2 and carbon coupling synergistically boost the activity of Ru nanoparticles for the alkaline hydrogen evolution reaction. *Journal of Materials Chemistry A.* **9**, 10160-10168 (2021).
40 Han S, Li X, Zeng X, Cao D, Chen J-F. Ru–MoS2@PPy hollow nanowire as an ultra-stable catalyst for alkaline hydrogen evolution reaction. *International Journal of Hydrogen Energy.* **47**, 37850-37859 (2022).
41 Wang Y*, et al.* Interfacial synergy between dispersed Ru sub-nanoclusters and porous NiFe layered double hydroxide on accelerated overall water splitting by intermediate modulation. *Nanoscale.* **12**, 9669-9679 (2020).
42 Shao X*, et al.* Amorphization of Metal Nanoparticles by 2D Twisted Polymer for Super Hydrogen Evolution Reaction. *Advanced Energy Materials.* **12**, 2102257 (2022).
43 Li J*, et al.* Green Electrosynthesis of 5,5′-Azotetrazolate Energetic Materials Plus Energy-Efficient Hydrogen Production Using Ruthenium Single-Atom Catalysts. *Advanced Materials.* **34**, 2203900 (2022).
44 Lao M*, et al.* Manipulating the Coordination Chemistry of Ru N(O) C Moieties for Fast Alkaline Hydrogen Evolution Kinetics. *Advanced Functional Materials.* **31**, 2100698 (2021).
45 Capozzoli L*, et al.* Ruthenium-loaded titania nanotube arrays as catalysts for the hydrogen evolution reaction in alkaline membrane electrolysis. *Journal of Power Sources.* **562**, 232747 (2023).
46 Wu W, Wu Y, Zheng D, Wang K, Tang Z. Ni@Ru core-shell nanoparticles on flower-like carbon nanosheets for hydrogen evolution reaction at All-pH values, oxygen evolution reaction and overall water splitting in alkaline solution. *Electrochimica Acta.* **320**, 134568

(2019).
47 Liu L*, et al.* Ru/MoO2 decorated on CNT networks as an efficient electrocatalyst for boosting hydrogen evolution reaction. *International Journal of Hydrogen Energy.* **47**, 26978-26986 (2022).
48 Hong C-B, Li X, Wei W-B, Wu X-T, Zhu Q-L. Nano-engineering of Ru-based hierarchical porous nanoreactors for highly efficient pH-universal overall water splitting. *Applied Catalysis B: Environmental.* **294**, 120230 (2021).
49 Li H*, et al.* Paired Ru–O–Mo ensemble for efficient and stable alkaline hydrogen evolution reaction. *Nano Energy.* **82**, 105767 (2021).
50 Liu J*, et al.* Visualizing spatial potential and charge distribution in Ru/N-doped carbon electrocatalysts for superior hydrogen evolution reaction. *Journal of Materials Chemistry A.* **7**, 18072-18080 (2019).
51 Zhao Y, Mao G, Huang C, Cai P, Cheng G, Luo W. Decorating WSe2 nanosheets with ultrafine Ru nanoparticles for boosting electrocatalytic hydrogen evolution in alkaline electrolytes. *Inorganic Chemistry Frontiers.* **6**, 1382-1387 (2019).
52 Khalid M*, et al.* Facile synthesis of Ru nanoclusters embedded in carbonaceous shells for hydrogen evolution reaction in alkaline and acidic media. *Journal of Electroanalytical Chemistry.* **929**, 117116 (2023).
53 Mo S, Zhou P, Li C, Liu J, Wang F. Atomic interface engineering: Strawberry-like RuO2/C hybrids for efficient hydrogen evolution from ammonia borane and water. *International Journal of Hydrogen Energy.* **46**, 22397-22408 (2021).
54 Liu G*, et al.* Synthesis of RuNi alloy nanostructures composed of multilayered nanosheets for highly efficient electrocatalytic hydrogen evolution. *Nano Energy.* **66**, 104173 (2019).
55 Wang Z*, et al.* Optimizing the Electronic Structure of Atomically Dispersed Ru Sites with CoP for Highly Efficient Hydrogen Evolution in both Alkaline and Acidic Media. *Small.* **19**, 2301403 (2023).
56 Zhu J*, et al.* Tensile-strained ruthenium phosphide by anion substitution for highly active and durable hydrogen evolution. *Nano Energy.* **77**, 105212 (2020).
57 Jiang Y*, et al.* Revealing and magnifying interfacial effects between ruthenium and carbon supports for efficient hydrogen evolution. *Journal of Materials Chemistry A.* **10**, 17730-17739 (2022).
58 Zhang H*, et al.* CD71-Specific Aptamer Conjugated with Monomethyl Auristatin E for the Treatment of Uveal Melanoma. *ACS Applied Materials & Interfaces.* **14**, 32-40 (2022).
59 Salah A*, et al.* Advanced Ru/Ni/WC@NPC Multi-Interfacial Electrocatalyst for Efficient Sustainable Hydrogen and Chlor-Alkali Co-Production. *Advanced Energy Materials.* **12**, 2200332 (2022).
60 Guo J-X*, et al.* High electrocatalytic hydrogen evolution activity on a coupled Ru and CoO hybrid electrocatalyst. *Journal of Energy Chemistry.* **37**, 143-147 (2019).
61 Sarkar B, Das D, Nanda KK. pH-dependent hydrogen evolution using spatially confined ruthenium on hollow N-doped carbon nanocages as a Mott–Schottky catalyst. *Journal of Materials Chemistry A.* **9**, 13958-13966 (2021).
62 Luo Q*, et al.* Synthesis of ultrafine ruthenium phosphide nanoparticles and nitrogen/phosphorus dual-doped carbon hybrids as advanced electrocatalysts for all-pH hydrogen evolution reaction. *International Journal of Hydrogen Energy.* **44**, 25632-25641

(2019).
63 Li X, Han S, Qiao Z, Zeng X, Cao D, Chen J. Ru monolayer island doped MoS_2 catalysts for efficient hydrogen evolution reaction. *Chemical Engineering Journal.* **453**, 139803 (2023).
64 Feng Y*, et al.* Reduced graphene oxide-supported ruthenium nanocatalysts for highly efficient electrocatalytic hydrogen evolution reaction. *International Journal of Hydrogen Energy.* **47**, 39853-39863 (2022).
65 Zhang S*, et al.* Supramolecular Anchoring Strategy for Facile Production of Ruthenium Nanoparticles Embedded in N-Doped Mesoporous Carbon Nanospheres for Efficient Hydrogen Generation. *ACS Applied Materials & Interfaces.* **13**, 32997-33005 (2021).
66 Wang Y*, et al.* In situ phase-reconfiguration to synthesize Ru, B co-doped nickel phosphide for energy-efficient hydrogen generation in alkaline electrolytes. *Journal of Materials Chemistry A.* **10**, 16236-16242 (2022).
67 Yu J*, et al.* Confining ultrafine Ru clusters into TiO_2 lattice frameworks to yield efficient and ultrastable electrocatalysts towards practical hydrogen evolution. *Chemical Engineering Journal.* **446**, 137248 (2022).
68 Wang Y, Luo W, Li H, Cheng C. Ultrafine Ru nanoclusters supported on N/S doped macroporous carbon spheres for efficient hydrogen evolution reaction. *Nanoscale Advances.* **3**, 5068-5074 (2021).
69 Fan X*, et al.* Synergistic effect of dual active sites over Ru/α-MoC for accelerating alkaline hydrogen evolution reaction. *Applied Catalysis B: Environmental.* **318**, 121867 (2022).
70 Chen D*, et al.* Ru-substituted Co nanoalloys encapsulated within graphene as efficient electrocatalysts for accelerating water dissociation in alkaline solution. *Applied Surface Science.* **580**, 152294 (2022).
71 Liu Z*, et al.* Engineering of Ru/Ru_2P interfaces superior to Pt active sites for catalysis of the alkaline hydrogen evolution reaction. *Journal of Materials Chemistry A.* **7**, 5621-5625 (2019).
72 Li S*, et al.* Geometric Structure and Electronic Polarization Synergistically Boost Hydrogen Evolution Kinetics in Alkaline Medium. *The Journal of Physical Chemistry Letters.* **11**, 3436-3442 (2020).
73 Gao T*, et al.* Understanding the Atomic and Defective Interface Effect on Ruthenium Clusters for the Hydrogen Evolution Reaction. *ACS Catalysis.* **13**, 49-59 (2023).
74 Luo J*, et al.* Efficient Contact between H_2O and N-Coordinate Ru Nanoparticles in Three-Dimensionally Ordered Macro/Mesoporous Carbon Boosting Alkaline HER†. *Chinese Journal of Chemistry.* **n/a**, (2023).
75 Huang H*, et al.* Surface conversion derived core-shell nanostructures of Co particles@RuCo alloy for superior hydrogen evolution in alkali and seawater. *Applied Catalysis B: Environmental.* **315**, 121554 (2022).
76 Chi J-Q*, et al.* Hydrogen Evolution Activity of Ruthenium Phosphides Encapsulated in Nitrogen- and Phosphorous-Codoped Hollow Carbon Nanospheres. *ChemSusChem.* **11**, 743-752 (2018).
77 Naseeb W, Liu Q, Nichols F, Pan D, Khosa MK, Chen S. Ru-CoO heterostructured nanoparticles supported on nitrogen and sulfur codoped graphene nanosheets as effective electrocatalysts for hydrogen evolution reaction in alkaline media. *Journal of Electroanalytical Chemistry.* **932**, 117272 (2023).

78 Peng Z, *et al.* Hollow carbon shells enhanced by confined ruthenium as cost-efficient and superior catalysts for the alkaline hydrogen evolution reaction. *Journal of Materials Chemistry A.* **7**, 6676-6685 (2019).
79 Zhang M, *et al.* Nitrogen-doped graphite encapsulating RuCo nanoparticles toward high-activity catalysis of water oxidation and reduction. *Chemical Engineering Journal.* **422**, 130077 (2021).
80 Luo T, *et al.* Fullerene Lattice-Confined Ru Nanoparticles and Single Atoms Synergistically Boost Electrocatalytic Hydrogen Evolution Reaction. *Advanced Functional Materials.* **33**, 2213058 (2023).
81 Liu Z, *et al.* Boosted hydrogen evolution reaction based on synergistic effect of RuO2@MoS2 hybrid electrocatalyst. *Applied Surface Science.* **538**, 148019 (2021).
82 Zhao Y, *et al.* Heterostructure of RuO2-RuP2/Ru Derived from HMT-based Coordination Polymers as Superior pH-Universal Electrocatalyst for Hydrogen Evolution Reaction. *Small.* **18**, 2105168 (2022).
83 Chen J, *et al.* Atomic ruthenium coordinated with chlorine and nitrogen as efficient and multifunctional electrocatalyst for overall water splitting and rechargeable zinc-air battery. *Chemical Engineering Journal.* **441**, 136078 (2022).
84 Liu N, Zhai Z, Yu B, Yang W, Cheng G, Zhang Z. Bifunctional nanoporous ruthenium-nickel alloy nanowire electrocatalysts towards oxygen/hydrogen evolution reaction. *International Journal of Hydrogen Energy.* **47**, 31330-31341 (2022).
85 Liu Y, *et al.* Self-crosslinking carbon dots loaded ruthenium dots as an efficient and super-stable hydrogen production electrocatalyst at all pH values. *Nano Energy.* **65**, 104023 (2019).
86 Wu Q, *et al.* Identifying Electrocatalytic Sites of the Nanoporous Copper–Ruthenium Alloy for Hydrogen Evolution Reaction in Alkaline Electrolyte. *ACS Energy Letters.* **5**, 192-199 (2020).
87 Li D, Zhang B, Li Y, Chen R, Hu S, Ni H. Boosting hydrogen evolution activity in alkaline media with dispersed ruthenium clusters in NiCo-layered double hydroxide. *Electrochemistry Communications.* **101**, 23-27 (2019).
88 Zhou S, *et al.* Ru atom-modified Co4N-CoF2 heterojunction catalyst for high-performance alkaline hydrogen evolution. *Chemical Engineering Journal.* **414**, 128865 (2021).
89 Chen C-H, *et al.* Ruthenium-Based Single-Atom Alloy with High Electrocatalytic Activity for Hydrogen Evolution. *Advanced Energy Materials.* **9**, 1803913 (2019).
90 Zhang Z, *et al.* Benchmarking Phases of Ruthenium Dichalcogenides for Electrocatalysis of Hydrogen Evolution: Theoretical and Experimental Insights. *Small.* **17**, 2007333 (2021).
91 Li J-S, *et al.* RuP2-based hybrids derived from MOFs: highly efficient pH-universal electrocatalysts for the hydrogen evolution reaction. *Journal of Materials Chemistry A.* **9**, 12276-12282 (2021).
92 Li H, *et al.* Ultrafine Ru nanoparticles confined in 3D nitrogen-doped porous carbon nanosheet networks for alkali-acid Zn-H2 hybrid battery. *Applied Catalysis B: Environmental.* **280**, 119412 (2021).
93 Yu H, *et al.* Interface engineering of Ni5P2 nanoparticles and a mesoporous PtRu film heterostructure on Ni foam for enhanced hydrogen evolution. *Nanotechnology.* **30**,

485403 (2019).
94 Li M, Wang H, Zhu W, Li W, Wang C, Lu X. RuNi Nanoparticles Embedded in N-Doped Carbon Nanofibers as a Robust Bifunctional Catalyst for Efficient Overall Water Splitting. *Advanced Science.* **7**, 1901833 (2020).
95 Kunitski M*, et al.* Double-slit photoelectron interference in strong-field ionization of the neon dimer. *Nature Communications.* **10**, 1 (2019).
96 Luo R*, et al.* Ultrafine Ru nanoparticles derived from few-layered Ti3C2Tx MXene templated MOF for highly efficient alkaline hydrogen evolution. *International Journal of Hydrogen Energy.* **47**, 32787-32795 (2022).
97 Tang L, Yu J, Zhang Y, Tang Z, Qin Y. Boosting the hydrogen evolution reaction activity of Ru in alkaline and neutral media by accelerating water dissociation. *RSC Advances.* **11**, 6107-6113 (2021).
98 Xia J, Volokh M, Peng G, Fu Y, Wang X, Shalom M. Low-Cost Porous Ruthenium Layer Deposited on Nickel Foam as a Highly Active Universal-pH Electrocatalyst for the Hydrogen Evolution Reaction. *ChemSusChem.* **12**, 2780-2787 (2019).
99 Thiyagarajan D, Thirumurugan A, Lee B-K. Efficient CoMoRu0.25Ox/NF nanoplate architectures for overall electrochemical water splitting. *International Journal of Hydrogen Energy.* **47**, 39908-39916 (2022).
100 Yoon D*, et al.* Cactus-Like Hollow Cu2-xS@Ru Nanoplates as Excellent and Robust Electrocatalysts for the Alkaline Hydrogen Evolution Reaction. *Small.* **13**, 1700052 (2017).
101 Ma Q*, et al.* Ultralow Ru-assisted and vanadium-doped flower-like CoP/Ni2P heterostructure for efficient water splitting in alkali and seawater. *Journal of Materials Chemistry A.* **9**, 26852-26860 (2021).
102 He Q*, et al.* Achieving Efficient Alkaline Hydrogen Evolution Reaction over a Ni5P4 Catalyst Incorporating Single-Atomic Ru Sites. *Adv. Mater.* **32**, 1906972 (2020).
103 Zhang X-Y*, et al.* In situ electro-oxidation modulation of Ru(OH)x/Ag supported on nickel foam for efficient hydrogen evolution reaction in alkaline media. *International Journal of Hydrogen Energy.* **44**, 21683-21691 (2019).
104 Qin L, Song T, Guo L, Huang K, Xue H, Wang Q. Boosting the electrocatalytic performance of ultrathin NiP2 nanosheets by synergic effect of W and Ru doping engineering. *Applied Surface Science.* **508**, 145302 (2020).
105 Meng G*, et al.* Ru to W electron donation for boosted HER from acidic to alkaline on Ru/WNO sponges. *Nano Energy.* **80**, 105531 (2021).
106 Chen H-Y*, et al.* Flower-like platinum-cobalt-ruthenium alloy nanoassemblies as robust and highly efficient electrocatalyst for hydrogen evolution reaction. *Journal of Colloid and Interface Science.* **561**, 372-378 (2020).
107 Moore GWK, Howell SEL, Brady M, Xu X, McNeil K. Anomalous collapses of Nares Strait ice arches leads to enhanced export of Arctic sea ice. *Nature Communications.* **12**, 1 (2021).
108 Liu X*, et al.* Charge Redistribution Caused by S,P Synergistically Active Ru Endows an Ultrahigh Hydrogen Evolution Activity of S-Doped RuP Embedded in N,P,S-Doped Carbon. *Advanced Science.* **7**, 2001526 (2020).
109 Li Y*, et al.* Partially exposed RuP2 surface in hybrid structure endows its bifunctionality for hydrazine oxidation and hydrogen evolution catalysis. *Science advances.* **6**, eabb4197

(2020).
110 Zhang L, *et al.* Exploring the Dominant Role of Atomic- and Nano-Ruthenium as Active Sites for Hydrogen Evolution Reaction in Both Acidic and Alkaline Media. *Adv. Sci.* . **8**, 2004516 (2021).
111 Liang Q, *et al.* Superassembly of Surface-Enriched Ru Nanoclusters from Trapping–Bonding Strategy for Efficient Hydrogen Evolution. *ACS Nano.* **16**, 7993-8004 (2022).
112 Wu Y, Wang L, Bo T, Chai Z, Gibson JK, Shi W. Boosting Hydrogen Evolution in Neutral Medium by Accelerating Water Dissociation with Ru Clusters Loaded on Mo2CTx MXene. *Advanced Functional Materials.* **33**, 2214375 (2023).
113 Zhao J, *et al.* Anchoring Ru nanoclusters to defect-rich polymeric carbon nitride as a bifunctional electrocatalyst for highly efficient overall water splitting. *Journal of Materials Chemistry A.* **11**, 18375-18386 (2023).
114 Da P, *et al.* Synthesis of Bandgap-tunable Transition Metal Sulfides through Gas-phase Cation Exchange - induced Topological Transformation. *Angewandte Chemie.* **135**, e202301802 (2023).
115 Samanta R, Mishra R, Barman S. Interface- and Surface-Engineered PdO−RuO2 Hetero-Nanostructures with High Activity for Hydrogen Evolution/Oxidation Reactions. *ChemSusChem.* **14**, 2112-2125 (2021).
116 Wang MJ, *et al.* Enhanced hydrogen evolution of single-atom Ru sites via geometric and electronic engineering: N and S dual coordination. *Applied Surface Science.* **551**, 148742 (2021).
117 Shen Q, *et al.* In-situ formed Cu-doped RuS2 hollow polyhedrons integrated with simultaneously heterostructure engineering with metallic Ru for boosting hydrogen evolution in alkaline media. *Materials Today Physics.* **23**, 100625 (2022).
